# Machine learning identifies PYGM as a macrophage polarization–linked metabolic biomarker in rectal cancer prognosis

**DOI:** 10.3389/fimmu.2025.1639303

**Published:** 2025-08-12

**Authors:** Chengyuan Xu, Siqi Zhang, Bin Sun, Zicheng Yu, Hailong Liu

**Affiliations:** ^1^ Department of General, Yangpu Hospital, School of Medicine, Tongji University, Shanghai, China; ^2^ Center for Clinical Research and Translational Medicine, Yangpu Hospital, School of Medicine, Tongji University, Shanghai, China; ^3^ Department of Pharmacy, Yangpu Hospital, School of Medicine, Tongji University, Shanghai, China

**Keywords:** rectal cancer, macrophage polarization, PYGM, metabolism, prognosis, machine learning

## Abstract

**Background:**

Macrophage polarization plays a pivotal role in shaping the tumor microenvironment and influencing rectal cancer progression. However, the metabolic and prognostic regulators governing this process remain largely undefined.

**Methods:**

We constructed a macrophage polarization gene signature (MPGS) by integrating weighted gene co-expression network analysis (WGCNA) with multiple machine learning algorithms across two independent cohorts: 363 rectal cancer samples from GSE87211 and 177 samples from The Cancer Genome Atlas (TCGA). The prognostic performance of MPGS was evaluated across rectal and multiple other cancer types. Functional analyses, single-cell RNA sequencing, immunohistochemistry of clinical specimens, and *in vitro* cellular assays were employed to investigate the role of the MPGS hub gene, *PYGM*, in tumor biology and immune modulation.

**Results:**

The MPGS exhibited robust prognostic capability and effectively predicted responses to immunotherapy and various chemotherapeutic agents. Both MPGS and its central metabolic component, *PYGM*, were closely linked to M2 macrophage infiltration, immunosuppressive tumor microenvironments, and poor clinical outcomes in rectal adenocarcinoma. Single-cell transcriptomic analysis revealed that malignant epithelial cells with elevated *PYGM* expression are metabolically active and closely interact with M2 macrophages. Clinical tissue analyses and functional assays confirmed that *PYGM* is upregulated in rectal cancer and promotes tumor cell proliferation, migration, and M2 macrophage polarization.

**Conclusions:**

This study firstly highlights *PYGM* as a key metabolic and immunological regulator in rectal cancer, with significant prognostic and therapeutic implications. MPGS and *PYGM* may serve as novel biomarkers for risk stratification and guide personalized treatment strategies in patients with rectal adenocarcinoma.

## 1 Introduction

Rectal cancer ranks as the eighth leading cause of cancer-related mortality globally, accounting for approximately 340,000 deaths in 2022 (1). While advances in high-resolution imaging and multimodal therapies—such as neoadjuvant chemoradiotherapy (nCRT), total mesorectal excision (TME), and organ-preserving strategies—have refined clinical management (2, 3), long-term outcomes remain suboptimal. Although colonoscopy is the gold standard for early detection, its high-cost limits widespread implementation in low- and middle-income countries (4). Rectal adenocarcinoma (READ) and colon adenocarcinoma (COAD), though both classified under colorectal cancer (CRC), exhibit distinct embryological origins, anatomical locations, treatment responses and clinical outcomes (5), molecular profiles (6), immune infiltration patterns (7, 8). The molecular pathogenesis of rectal cancer involves diverse genetic and epigenetic alterations, including dysregulation of genes such as APC, KRAS, TP53, MSI, SOCS2, and SOCS6 (9–11). Nonetheless, current biomarkers and therapeutic targets have limited utility, and compared to CRC as a whole, there is currently a relative scarcity of studies that specifically focus on rectal adenocarcinoma (READ) as an independent entity. Most existing prognostic models and tumor microenvironment analyses have been developed based on combined CRC cohorts, potentially overlooking the unique biological, molecular, and clinical characteristics of rectal cancer. There is a critical need to identify novel molecular determinants that can improve diagnostic precision and prognostic stratification in READ.

Tumor-associated macrophages (TAMs), particularly those with an M2-like polarization phenotype, are key immunosuppressive components of the tumor microenvironment (TME) and facilitate tumor progression by secreting pro-tumorigenic mediators such as CHI3L1 and TGF-β (12–14). TAMs play essential roles in modulating immune–tumor interactions, promoting angiogenesis, metastasis, and resistance to therapy (15–17). Phenotypically, TAMs resemble alternatively activated (M2) macrophages linked to poor clinical outcomes across multiple malignancies, including colorectal cancer (14, 17–22). In rectal cancer specifically, several studies have reported that individual gene alterations may affect macrophage infiltration and correlate with adverse prognosis (23, 24). However, these investigations are often limited to single-gene associations, lacking integrative modeling approaches that account for the complex regulatory landscape of macrophage polarization.

There is growing recognition that metabolic reprogramming in the TME fuels tumor cell proliferation and shapes the immune landscape, particularly by modulating macrophage differentiation and polarization (25–28). Tumor-driven lipid and glucose metabolism alterations generate a metabolically enriched and immunosuppressive environment that favors M2 macrophage accumulation. For instance, overexpression of sterol regulatory element-binding proteins (SREBPs) enhances lipid biosynthesis, contributing to M2 polarization via endoplasmic reticulum stress pathways (29, 30). Similarly, mitochondrial dysfunction, such as PINK1 deficiency, induces the Warburg effect in gastric cancer cells and promotes M2 macrophage recruitment (31). These findings underscore the potential of metabolic genes as dual-function biomarkers—informative of both macrophage activity and tumor progression.

In this study, we curated macrophage polarization–related genes from the GeneCards database and analyzed gene expression profiles from TCGA and GEO datasets (GSE87211 and others) to identify dysregulated genes associated with prognosis in rectal cancer. Through a combination of weighted gene co-expression network analysis (WGCNA) and four machine learning algorithms, we constructed a macrophage polarization gene signature (MPGS) and validated its prognostic utility across multiple independent cohorts. Functional enrichment, single-cell transcriptomic profiling, clinical sample validation and cell assays were performed to elucidate the biological role of the signature’s key component, *PYGM*, in metabolic regulation and macrophage infiltration. Our findings suggest that *PYGM* is a clinically relevant metabolic biomarker associated with immune modulation and survival outcomes in rectal cancer.

## 2 Materials and methods

### 2.1 Data acquisition and processing

Gene expression datasets (GSE87211, GSE14333, GSE117536, GSE17537, GSE17538, GSE38832, and GSE103479) were obtained from the Gene Expression Omnibus (GEO; https://www.ncbi.nlm.nih.gov/geo/) as of October 2023. Single-cell RNA sequencing (scRNA-seq) data were retrieved from GEO accession GSE132465. Bulk RNA-seq expression profiles and associated clinical data for rectal adenocarcinoma (READ) and colon adenocarcinoma (COAD) patients were acquired from The Cancer Genome Atlas (TCGA; https://portal.gdc.cancer.gov/). The TCGA pan-cancer dataset, comprising over 10,000 samples across 33 cancer types, was also included for external validation. A total of 10,598 macrophage polarization-related genes (MPGs) were retrieved from GeneCards (https://www.genecards.org/) using the keyword “macrophage polarization.” Mutation status of candidate genes was assessed using cBioPortal (https://www.cbioportal.org/) (32). Protein-level expression data of MPGs in normal and tumor tissues were accessed from the Human Protein Atlas (https://www.proteinatlas.org/) (33). Gene expression matrices were normalized using the NormalizeBetweenArrays function from the limma R package, and batch effect adjustment was performed using the “ComBat” algorithm from the sva package, with default parameters, to correct for potential batch effects across datasets. GSE87211 was designated the training cohort, while the TCGA-READ dataset served as the test cohort.

### 2.2 WGCNA for co-expression network construction

Weighted gene co-expression network analysis (WGCNA) was performed on the GSE87211 dataset using the WGCNA R package (34). An optimal soft-thresholding power (β) was selected to ensure scale-free network topology (power = 6, minimum module size = 30, with a module merging threshold of 0.25). An adjacency matrix was constructed and transformed into a topological overlap matrix (TOM) to measure gene connectivity. Genes were hierarchically clustered based on TOM dissimilarity, and distinct gene modules were identified using average linkage clustering. Module–trait correlations were calculated to identify modules most associated with clinical traits in the GSE87211 cohort. These modules were prioritized for downstream analysis.

### 2.3 Differential expression analysis

Differential expression analysis was conducted using the limma package (35) in R. Genes with |log2FoldChange| ≥ 0.5, and adjusted p-value< 0.05 were considered differentially expressed. Differentially expressed genes (DEGs) were intersected with the curated macrophage polarization genes (MPGs) to identify a subset of differentially expressed MPGs (DEMPGs) relevant to rectal cancer.

### 2.4 Survival analysis and machine learning for hub gene selection

Univariate Cox regression was performed to identify DEGs significantly associated with overall survival in the training and validation cohorts. Candidate hub genes were identified by intersecting WGCNA module genes, DEGs, and MPGs with prognostic significance. To refine this gene set, four machine learning algorithms—LASSO Cox regression (glmnet package), support vector machine (SVM; conducted using the “e1071” R package; Kernel function: Recursive Feature Elimination (RFE kernel)) (36), random forest (RF; 500 trees with default settings from the “randomForest” R package), and extreme gradient boosting (XGBoost; xgboost package) (37)—were applied. Genes selected by all four methods were defined as core DEMPGs for further modeling. The cross-validation strategy for machine learning models: 10-fold cross-validation, repeated 3 times to ensure model stability, using the “caret” R package

### 2.5 Construction and validation of the macrophage polarization signature

Multivariate Cox regression was applied to the core DEMPGs to construct a macrophage polarization gene signature (MPGS). The risk score for each patient was calculated as:


$$Risk\;Score=\sum_{t=1}^{n}\beta i\chi i$$

where βi represents the regression coefficient, and χi is the normalized expression value (FPKM) of each signature gene. Patients were stratified into high- and low-risk groups based on the median risk score. Kaplan–Meier survival curves and multivariate Cox models were used to evaluate the prognostic significance of MPGS, adjusting for clinical covariates. Receiver operating characteristic (ROC) curves were generated using the timeROC package (38), and calibration curves were plotted to compare predicted and observed survival. Validation was performed in the independent TCGA-READ dataset.

### 2.6 Functional and immune infiltration analysis

DEGs between high- and low-risk groups (|log2FoldChange| ≥ 0.5, and adjusted p-value< 0.05) were identified using limma. Gene ontology (GO), Kyoto Encyclopedia of Genes and Genomes (KEGG), and gene set enrichment analysis (GSEA) were conducted using the clusterProfiler package (39) to explore biological processes and pathways enriched in the high-risk group, false discovery rate (FDR)< 0.5 and normalized enrichment score (NES) > 1 were set at the cut-off criteria. Immune cell composition and tumor microenvironment (TME) scores were assessed using the CIBERSORT, QuanTIseq, and single-sample GSEA (ssGSEA) algorithms.

### 2.7 Protein-protein interaction network analysis

Protein-protein interaction (PPI) networks for hub genes were constructed using GeneMANIA (http://www.genemania.org) (40, 41), an integrative platform that incorporates data on co-expression, physical interaction, co-localization, and functional annotations. Functional enrichment analysis was conducted to identify biological processes and pathways potentially regulated by the candidate genes.

### 2.8 Drug sensitivity prediction and TIDE, IPS scores

Drug response analysis was performed using the prophetic and oncoPredict R packages (42, 43). Half-maximal inhibitory concentration (IC50) values for various chemotherapeutic agents were predicted and correlated with the MPGS risk scores. Differences in drug sensitivity between risk groups were visualized using scatter plots, highlighting drugs with significant IC50 variation. The TIDE scores were calculated utilizing the Tumor Immune Dysfunction and Exclusion (TIDE, http://tide.dfci.harvard.edu/login/) database (44, 45). Moreover, immunophenoscore (IPS) of GC patients were obtained in The Cancer Immunome Atlas (TCIA, https://tcia.at/home) database (46).

### 2.9 Single-cell RNA-seq data processing

Single-cell RNA-seq data were processed using Seurat v4.3.0 for quality control, normalization, and dimensionality reduction. Cells with fewer than 400 genes or mitochondrial content exceeding 20% were excluded. Doublets were removed using DoubletFinder v2.0.3. Integration across samples was performed with Harmony v1.2.3. Principal component analysis (PCA) and Uniform Manifold Approximation and Projection (UMAP) were used for dimensionality reduction and clustering and the top 30 PCs were retained for downstream analysis. Downstream analyses were based on integrated expression matrices.

### 2.10 Cell type identification

Cell type annotation was performed using the Seurat FindAllMarkers function to identify cluster-specific marker genes (adjusted p< 0.05, min.pct > 0.25, |log2FC| > 0.25). Initial annotations were derived using the SingleR package and cross-validated against the CellMarker database. Manual curation was performed to confirm annotations based on canonical gene expression profiles from the literature.

### 2.11 Pathway enrichment analysis of metabolic signatures

Fifty hallmark gene sets were downloaded from the Molecular Signatures Database (MSigDB v7.5.1; https://www.gsea-msigdb.org/gsea/msigdb) (47) Metabolic activity scores were calculated at the single-cell level based on the mean scaled expression of all genes within each signature, as previously described (48). Differential expression of pathway scores between tumor and normal tissues was assessed using the FindAllMarkers function in Seurat, with an adjusted p-value threshold of< 0.05.

### 2.12 The chromosomal copy−number variations estimation

Chromosomal copy number variations (CNVs) were inferred using the R package “inferCNV”. Epithelial cells in normal tissues served as reference populations. For each cell subcluster, CNV scores were calculated by aggregating the CNV levels of all constituent cells. The threshold parameter was set to 0.1, while other settings remained at default.

### 2.13 Cell–cell communication analysis

Cell–cell communication analysis was carried out using the R package “CellChat” (version 1.1.3). To ensure consistent sampling across cell subclusters, 500 cells were randomly selected from each subpopulation using the subset function. The analysis incorporated three major signaling categories from the CellChat database: Secreted Signaling, ECM–Receptor, and Cell–Cell Contact. A minimum threshold of 10 cells per cluster was applied to filter out low-abundance populations (49).

### 2.14 Clinical samples and ethical approval

A total of 40 paired rectal adenocarcinoma (READ) and adjacent normal tissue samples were collected from patients undergoing surgical resection at Yangpu Hospital, Tongji University, between November 2018 and November 2019. All procedures were approved by the Ethics Committee of Yangpu Hospital (Approval No. LL-2023-LW-012). Fresh specimens were fixed in 4% paraformaldehyde for immunohistochemistry and snap-frozen in liquid nitrogen for RNA and protein extraction.

### 2.15 Quantitative real-time PCR and Western blotting

Total RNA was extracted from paired tumor and adjacent tissues using TRIzol reagent and reverse-transcribed into cDNA using a commercial kit (Takara, Dalian, China). Quantitative real-time PCR (qRT-PCR) was performed using gene-specific primers (**Supplementary Table 1**).

For protein extraction, tissues were lysed in RIPA buffer (Solarbio, China) with protease inhibitors (1:100, Thermo Scientific). Western blotting used the following primary antibodies: PYGM (1:1,000; ProteinTech, 19716-1-AP), β-actin (1:4,000; ProteinTech, 66009-1-Ig), Arg1(1:1,000; ProteinTech, 16001-1-AP), CD301(1:1,000; ProteinTech, 13590-1-AP), CD206(1:1,000; ProteinTech, 32647-1-AP), IL-10(1:1,000; ProteinTech, 60269-1-Ig).

### 2.16 Immunohistochemistry

Formalin-fixed, paraffin-embedded tissue blocks were sectioned at 4 μm thickness. Sections were dewaxed, rehydrated, and subjected to antigen retrieval using a pressure cooker for 30 minutes. Endogenous peroxidase activity was blocked using 3% hydrogen peroxide for 20 minutes. Non-specific binding was minimized with 5% BSA for 40 minutes. Sections were incubated overnight at 4°C with anti-PYGM primary antibody (1:100; ProteinTech, 19716-1-AP). Visualization was achieved using a DAB detection kit and counterstaining with hematoxylin.

### 2.17 Cell culture and transfection

Three human colorectal cancer (CRC) cell lines—HCT116, LOVO, and SW620—and the normal colonic epithelial cell line NCM460 were obtained from the Shanghai Institute of Biochemistry and Cell Biology. All cell lines were maintained in DMEM supplemented with 10% fetal bovine serum (FBS; Gibco, Carlsbad, CA, USA) at 37°C in a humidified incubator with 5% CO_2_. THP-1 cells were cultured in RPMI-1640 medium (Gibco) supplemented with 10% fetal bovine serum (FBS). Lipofectamine 3000 (Invitrogen, Carlsbad, CA, USA) was used to transfect cells with an siRNA specific for *PYGM* and a control construct purchased from GeneChem (Shanghai, China) (**Supplementary Table 2**). Cells were utilized for downstream assays at 48h post-transfection. Analyses were conducted in triplicate. *PYGM* overexpression plasmid was customized from GenePharma (Shanghai, China).

### 2.18 Transwell and wound healing assays

Migration and invasion assays were performed using 24-well Transwell chambers (Nest, China). Cells were seeded in serum-free DMEM (250μL) into the upper chamber, and 600μL of DMEM with 10% FBS was added to the lower chamber. For invasion assays, inserts were pre-coated with Matrigel (2mg/mL). After 24hours, non-migrated/invaded cells were removed, and cells on the lower membrane surface were fixed with 4% paraformaldehyde and stained with crystal violet for 10 minutes. Cells were quantified in five non-overlapping fields under a microscope (Nikon, Japan).

Confluent cells were scratched using a 10 μL pipette tip for the wound healing assay and cultured in a serum-free medium. Images were taken at 0 and 24hours using phase-contrast microscopy to assess wound closure.

### 2.19 Assessment of cell proliferation and M2 macrophage polarization

To assess the rate of DNA synthesis, CRC cell lines were subjected to treatment with 5-ethynyl-2’-deoxyuridine (EDU) at a concentration of 50 μM, which was subsequently added to the cell culture plates. Following a 30-minute incubation, DNA was stained using Hoechst 33342, allowing for the visualization of positively stained cells under a microscope. HCT116 and SW620 cells, characterized by either *PYGM* overexpression or knockdown, were dissociated into single-cell suspensions using 0.25% trypsin. These cells were then stained with Annexin V-APC and 7-Aminoactinomycin D (7-AAD) to evaluate apoptosis rates. THP-1 monocytes were first differentiated into macrophage-like cells by treatment with 200 ng/mL phorbol 12-myristate 13-acetate (PMA) for 48 hours. Following differentiation, the PMA-treated THP-1 cells were gently washed with PBS to remove residual PMA and were then seeded into the chamber of the transwell system for indirect co-culture.

After 48 hours of co-culture, THP-1-derived macrophages were collected and stained with anti-CD301-APC and anti-CD206-APC, and the number of CD301 or CD206-positive cells in macrophages was analyzed by flow cytometry. Meanwhile, total RNA and protein of THP-1-derived macrophages were extracted for the detection of M2 macrophage markers.

### 2.20 Statistical analysis

All statistical analyses and visualizations were performed in R (v4.2.1). Visualization packages included ggplot2, ggpubr, and enrichplot. For comparisons between groups, the Wilcoxon rank-sum test was applied. A two-sided p-value< 0.05 was considered statistically significant.

## 3 Results

### 3.1 Identification of prognostically relevant modules and hub genes

**Figure 1:**
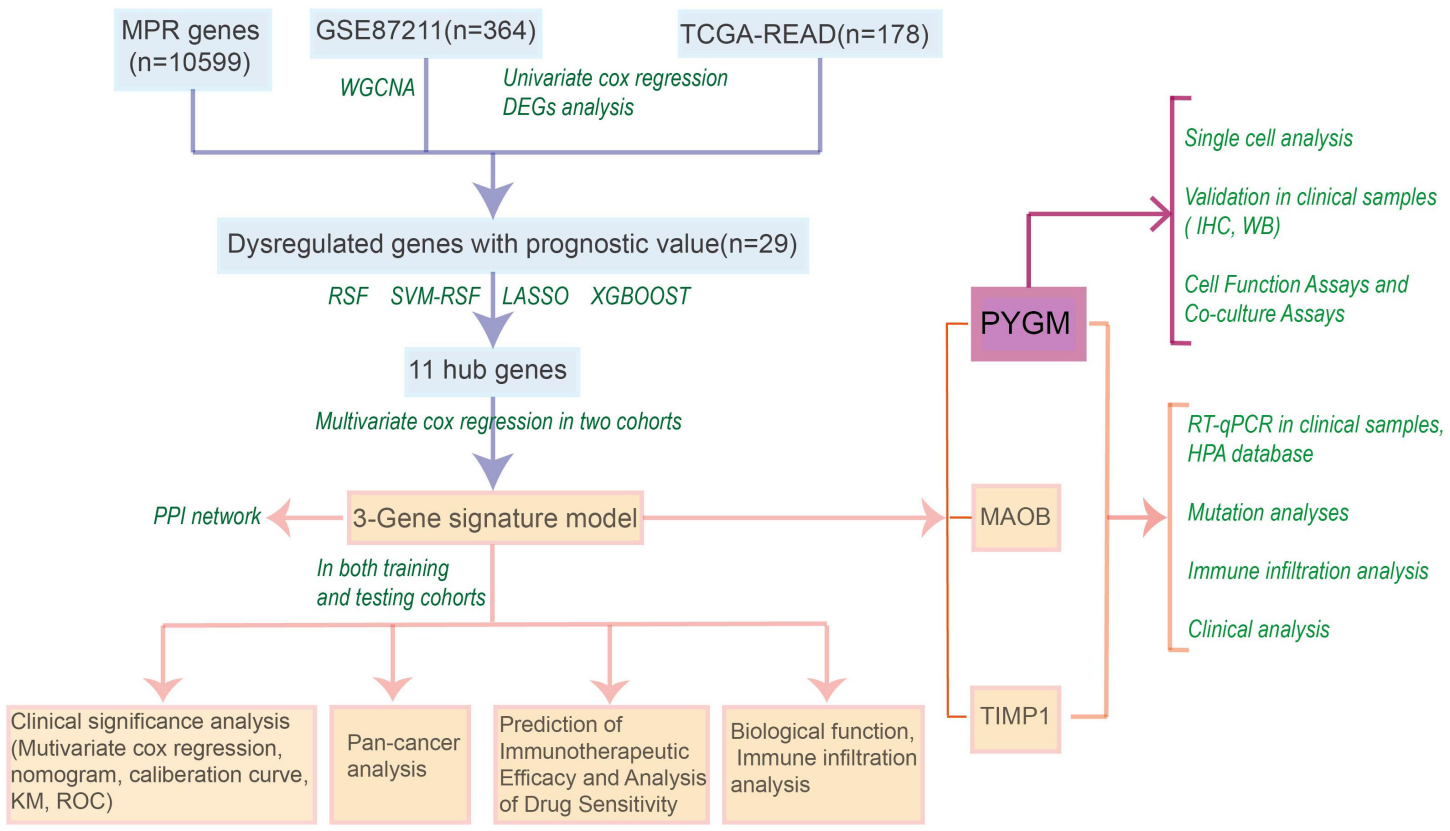
Flow chart of the manuscript.

**Figure 2:**
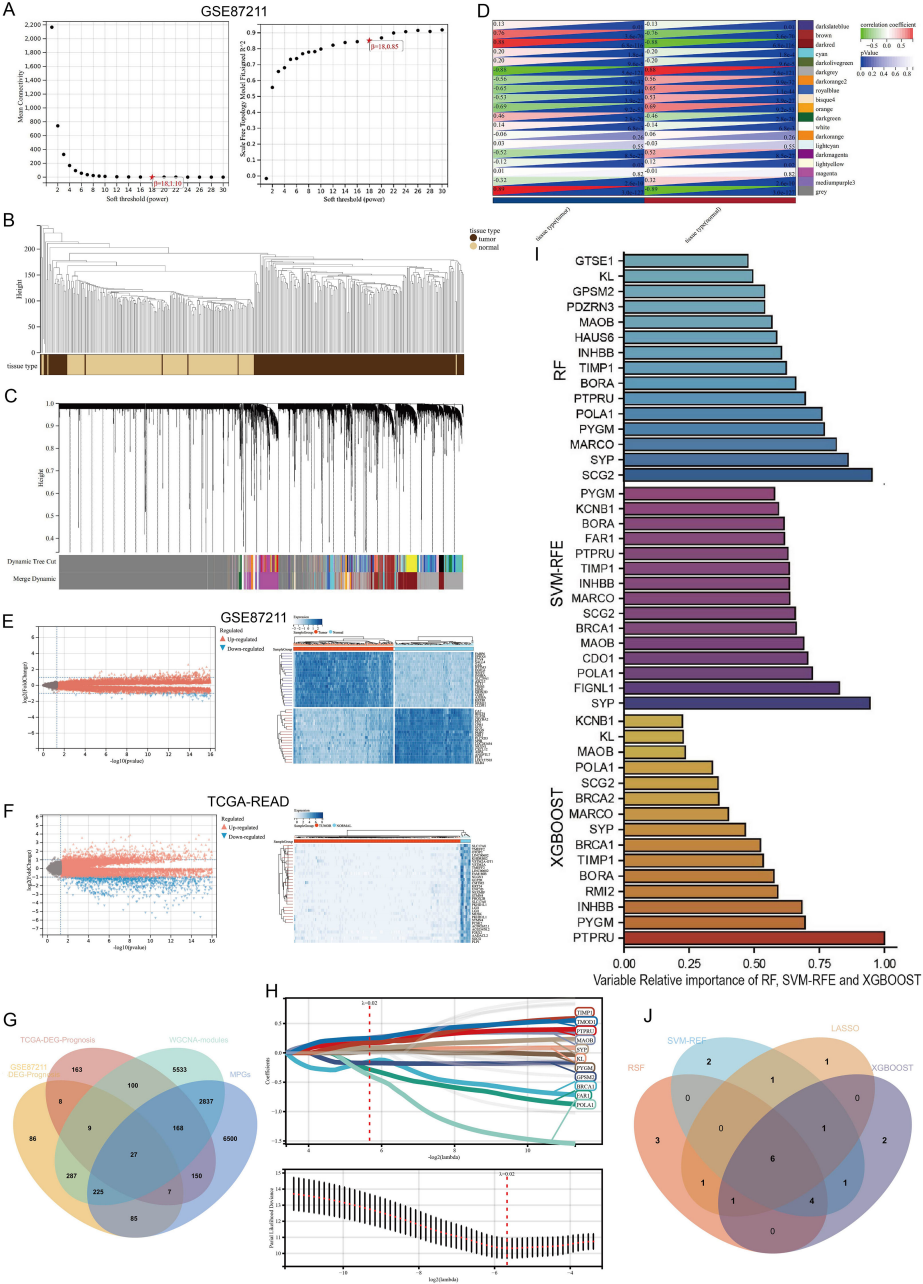
Construction of gene co-expression network and identification of hub DEGs. **(A)** Analysis of the scale-free fit index for various soft-thresholding powers (β). and the mean connectivity for various soft-thresholding powers. **(B)** Gene modules with different expression patterns. **(C)** Dendrogram of all differentially expressed genes clustered based on a dissimilarity measure (1-TOM). **(D)** Heatmap of the correlation between module eigengenes and clinical traits of rectal cancer. **(E, F)** Volcano plot and heat map of the differentially expressed genes in GSE87211 and TCGA datasets. **(G)** Overlap of DEGs associated with macrophage polarization, prognosis and WGCNA hub genes. **(H)** Optimal parameter (lambda) selection and coefficient distribution for LASSO models of 11 prognostic related genes. **(I)** Top 15 genes selected based on relative importance of RF, SVM-RFE and XGBOOST. **(J)** Venn diagram showing crossover genes after the analyses of XGBoost, RF, SVM-RFE and LASSO.

The workflow for model construction and downstream analyses is summarized in **Figure 1**. Weighted Gene Co-expression Network Analysis (WGCNA) was applied to the GSE87211 cohort to explore gene modules associated with rectal cancer. A soft-thresholding power of β = 18 was selected to ensure scale-free network topology (**Figure 2A**). Gene clustering yielded multiple expression modules, visualized via a dendrogram, each represented by a distinct color (**Figures 2B, C**). Among them, the dark red, dark grey, and brown modules demonstrated the strongest correlations with clinical traits (Pearson’s r = 0.88, –0.88, and 0.76, respectively; **Figure 2D**). The grey module, comprising unassigned genes, was excluded. Differential expression analysis identified 3,555 downregulated and 3,750 upregulated genes in the GSE87211 cohort; integration with TCGA-READ yielded 5,961 downregulated and 356 upregulated genes, visualized as volcano plots and heatmaps (**Figures 2E, F**).

Univariate Cox regression revealed 734 and 632 survival-associated genes in the GEO and TCGA datasets. By intersecting prognostic DEGs, WGCNA-derived module genes, and macrophage polarization genes (MPGs) from GeneCards, 29 candidate genes were identified (**Figure 2G**).

LASSO regression narrowed this set to 11 genes (*BRCA1, FAR1, GPSM2, KL, MAOB, POLA1, PTPRU*, *PYGM*, SYP, TIMP1, TMOD1; **Figure 2H**). Three additional machine learning methods—XGBoost, SVM-RFE, and Random Survival Forest (RSF)—identified the top 15 genes by feature importance. The intersection with LASSO output resulted in six shared hub genes (**Figures 2I, J**).

### 3.2 Development of a prognostic macrophage polarization gene signature

Multivariate Cox regression was performed on the six hub genes to refine the candidate genes. Three genes—*TIMP1, MAOB*, and *PYGM*—remained significant (p< 0.05) in both the GSE87211 and TCGA-READ cohorts (**Supplementary Figures S1A–S1F**).

These genes formed the basis of a prognostic risk model (MPGS), and the following formula was derived:


Risk Score=(0.33082×PYGM)+(0.43159×TIMP1)+(0.54156 ×MAOB)

**Figure 3:**
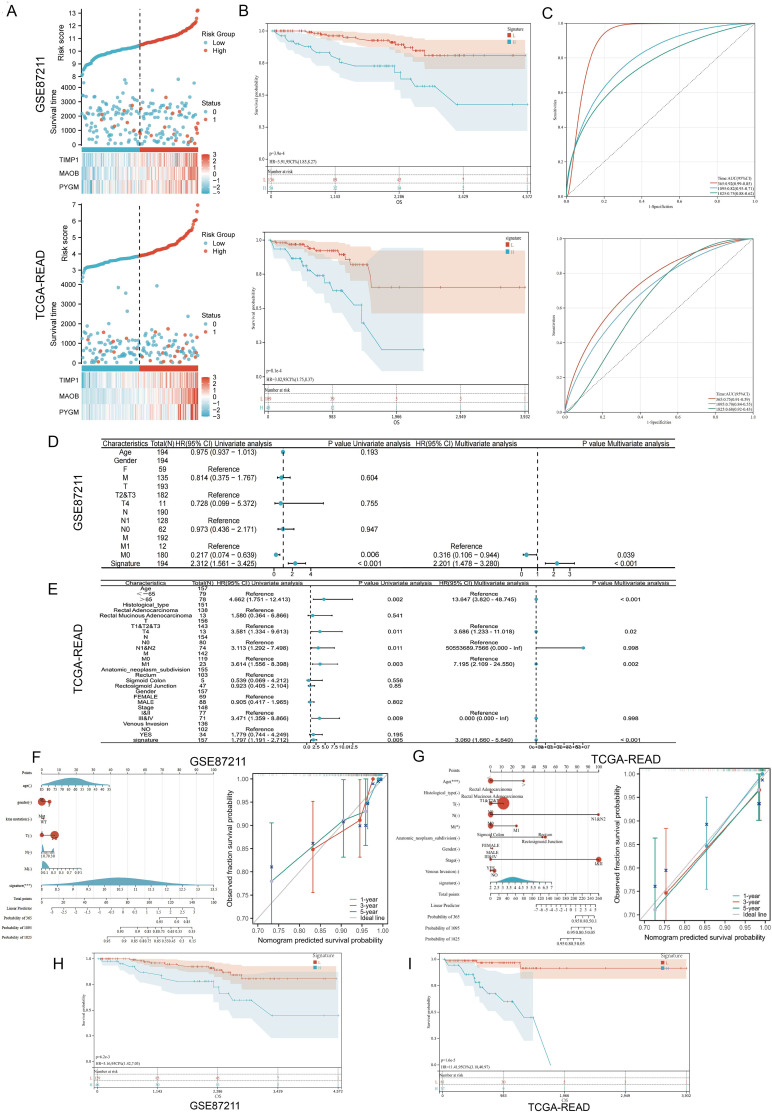
Validation of the prognostic signature. **(A)** Distribution of MPGs model based on risk score for the GSE87211 and TCGA cohorts, patterns of the survival time, and survival status between the high- and low-risk groups for the GSE87211 and TCGA set and clustering analysis heatmap shows the display levels of the three MPGs for each patient. **(B)** Kaplan–Meier survival curves of the OS of patients in the high- and low-risk cohorts for the two datasets. **(C)** Time-dependent ROC analysis of accuracy of the model in two datasets. **(D, E)** Univariate and multivariate Cox regression analyses in the GSE87211 and TCGA set. **(F, G)** Nomograms and calibration curves in 1-, 3-, and 5-year calibration curves according to signature expression. **(H, I)** Survival analysis of M0 subgroups in two datasets.

Patients in both cohorts were stratified into high- and low-risk groups based on the median risk score. Risk score distribution and corresponding survival status are displayed in **Figure 3A**, and gene expression heatmaps showed upregulation of all three genes in the high-risk group. Kaplan–Meier analysis confirmed that low-risk patients had significantly better overall survival (OS) in both datasets (**Figure 3B**). Receiver Operating Characteristic (ROC) analysis demonstrated good predictive performance of the MPGS for OS in both training and validation cohorts (**Figure 3C**).

### 3.3 Prognostic independence and stratification analyses

Univariate and multivariate Cox regression analyses were conducted to evaluate whether the MPGS was an independent predictor of OS. In the GSE87211 dataset, both MPGS (p<0.001) and M (P=0,006) stage were significantly associated with OS in univariate analysis, and MPGS remained independently prognostic in the multivariate model (**Figure 3D**). Similar findings were confirmed in the TCGA-READ cohort (**Figure 3E**).

A prognostic nomogram integrating MPGS and clinical variables was developed to predict 1-, 3-, and 5-year survival probabilities. Calibration curves demonstrated good agreement between predicted and observed outcomes in both cohorts (**Figures 3F, G**). Stratified survival analysis within M-stage subgroups revealed that high-risk patients in the M0 group exhibited significantly poorer OS than low-risk patients, while no significant difference was observed in the M1 subgroup (**Figures 3H–I**, **Supplementary Figure S2**). Additionally, both datasets’ Kaplan–Meier and ROC analyses showed that *PYGM* and *MAOB* exhibited strong diagnostic and prognostic performance (**Supplementary Figure S3**).

### 3.4 Functional enrichment and immune infiltration analyses

**Figure 4:**
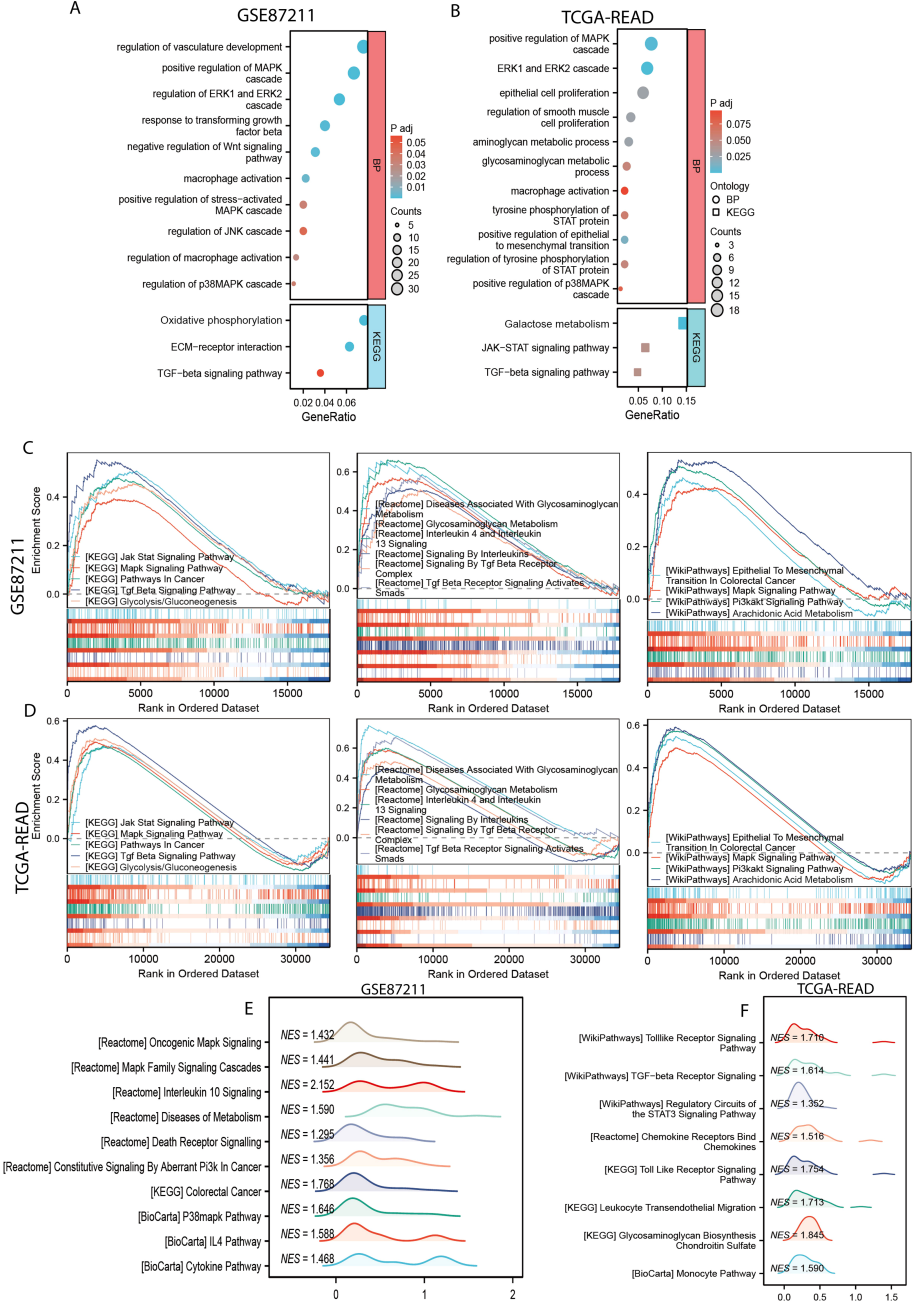
Functional enrichment analysis. **(A, B)** GO and KEGG enrichment analyses of the of differentially expressed genes (DEGs) between the high- and low-risk subgroups in GSE87211 and TCGA-READ dataset. **(C, D)** The common gene related with MPGS enriched in pathways in KEGG, Reactome, and WikiPathway databases in the GSE87211 and TCGA cohorts. **(E, F)** Unique pathways enriched in the GSE87211 and TCGA datasets.

To investigate the biological implications of the MPGS, Gene Ontology (GO) and Kyoto Encyclopedia of Genes and Genomes (KEGG) enrichment analyses were conducted. GO analysis highlighted immune- and metabolism-related processes, including positive regulation of the MAPK cascade, macrophage activation, and epithelial cell proliferation. KEGG pathway analysis further identified enrichment in the TGF-β signaling pathway, oxidative phosphorylation, and other metabolism-associated pathways in both cohorts (**Figures 4A, B**).

GSEA showed that the genes in the high-risk group from both cohorts were significantly enriched in several hallmark pathways, including Jak-Stat signaling pathway, MAPK signaling pathway (KEGG), glycosaminoglycan metabolism, interleukin 4 and interleukin 13 signaling (Reactome), epithelial-to-mesenchymal transition in colorectal cancer, and the PI3K-AKT signaling pathway (WikiPathways) (**Figures 4C, D**). Additionally, several other pathways related to cancer progression, macrophage polarization, and metabolism were enriched in the GSE87211 and TCGA cohorts, respectively (**Figures 4E, F**).

### 3.5 Immune infiltration, immunotherapy and drug sensitivity analysis

**Figure 5:**
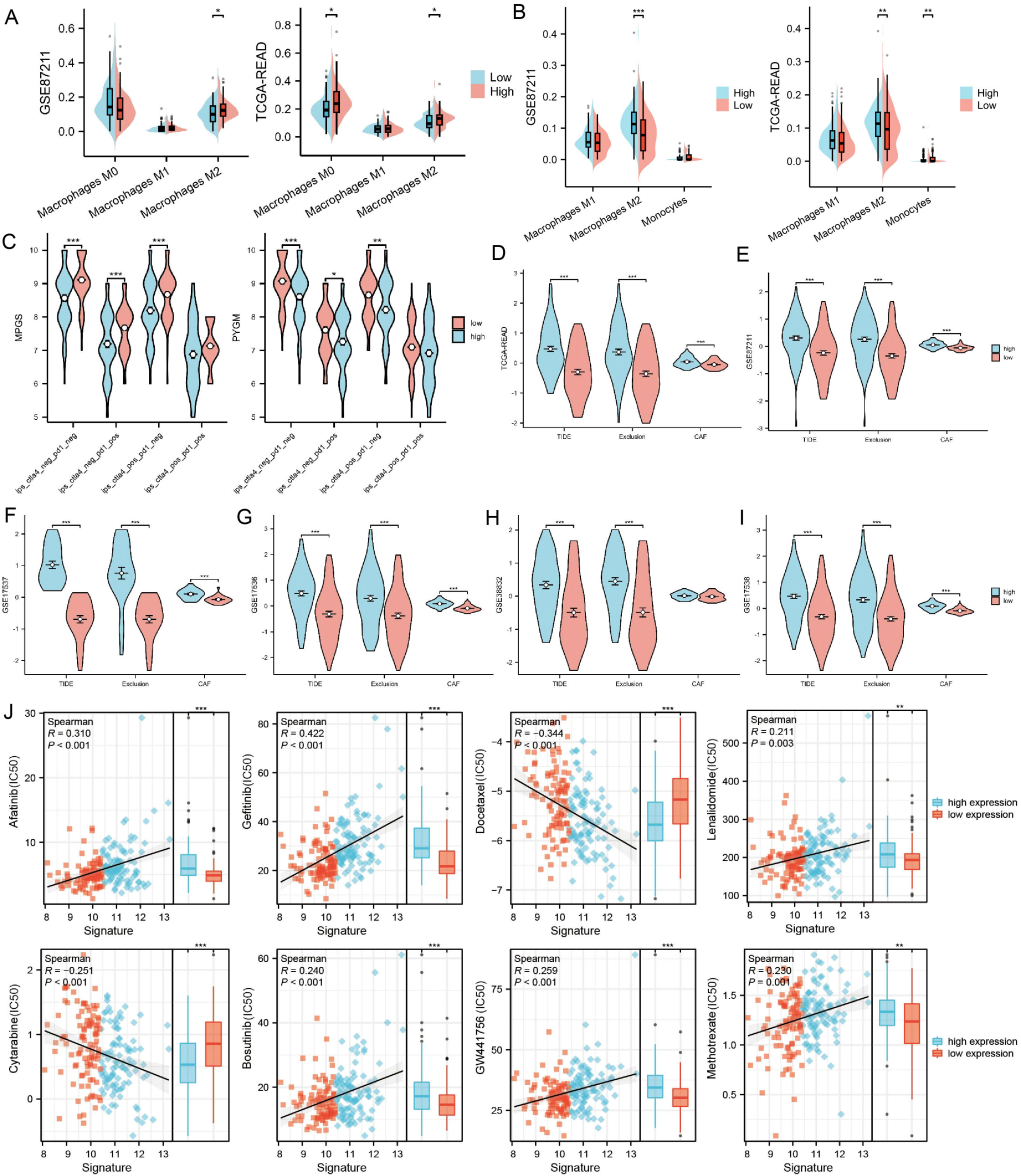
The association between PYGM expression and immune infiltration, immunotherapy response, and drug sensitivity prediction **(A, B)** Differences in tumor immune-infiltrating cell abundance between high- and low-risk groups were analyzed using the CIBERSORT and QuanTIseq algorithm. **(C)** Correlation of IPS with MPGS and PYGM expression. **(D–I)** Correlation of MPGSexpression with TIDE, exclusion and CAF in TCGA **(D)**, GSE87211 **(E)**, GSE17537 **(F)**, GSE17536 **(G)**, GSE38832**(H)**, GSE17538 **(I)** datasets. **(J)** Chemotherapy and immunotherapy sensitivity prediction between the low-risk and the high-risk groups. *p< 0.05; **p< 0.01; ***p< 0.001 compared to the corresponding groups.

Immune infiltration in the tumor microenvironment (TME) was assessed using CIBERSORT and QuanTIseq algorithms, revealing that M2 macrophages were enriched in the high-risk group (**Figures 5A, B**)

We analyzed the IPS of patients stratified by MPGS (**Figure 5C**, **Supplementary Figures S4A, B**) and the expression levels of three core genes using data from the TCIA database. The results showed that patients with low MPGS and low *PYGM* expression exhibited significantly higher IPS values compared to those with high expression levels, suggesting that lower MPGS and *PYGM* expression may be associated with improved responsiveness to immunotherapy.

Subsequently, multiple datasets were analyzed using the TIDE algorithm to evaluate the immunotherapy response between high- and low-MPGS expression groups. In the TIDE model, higher scores indicate a greater likelihood of immune evasion and a lower probability of benefiting from immune checkpoint inhibitor (ICI) therapy (44). The analysis revealed that the high-MPGS group exhibited significantly higher TIDE and Exclusion scores (**Figures 5D–I**), suggesting reduced sensitivity to immunotherapy. Consistently, *PYGM* showed a similar trend in both the training and validation cohorts (**Supplementary Figures S4C, D**).

Drug sensitivity prediction using the oncoPredict and prophetic packages indicated that IC50 values for Cytarabine and Docetaxel were significantly lower in high-risk patients, suggesting higher drug sensitivity while Lenalidomide, GW441756, Bosutinib, Afatinib, Gefitinib, Methotrexate, and GW441756 shown the opposite trend (**Figure 5J**). These findings may inform patient stratification and personalized chemotherapy, pending clinical validation.

### 3.6 Prognostic evaluation of MPGS across multiple cancer types

**Figure 6:**
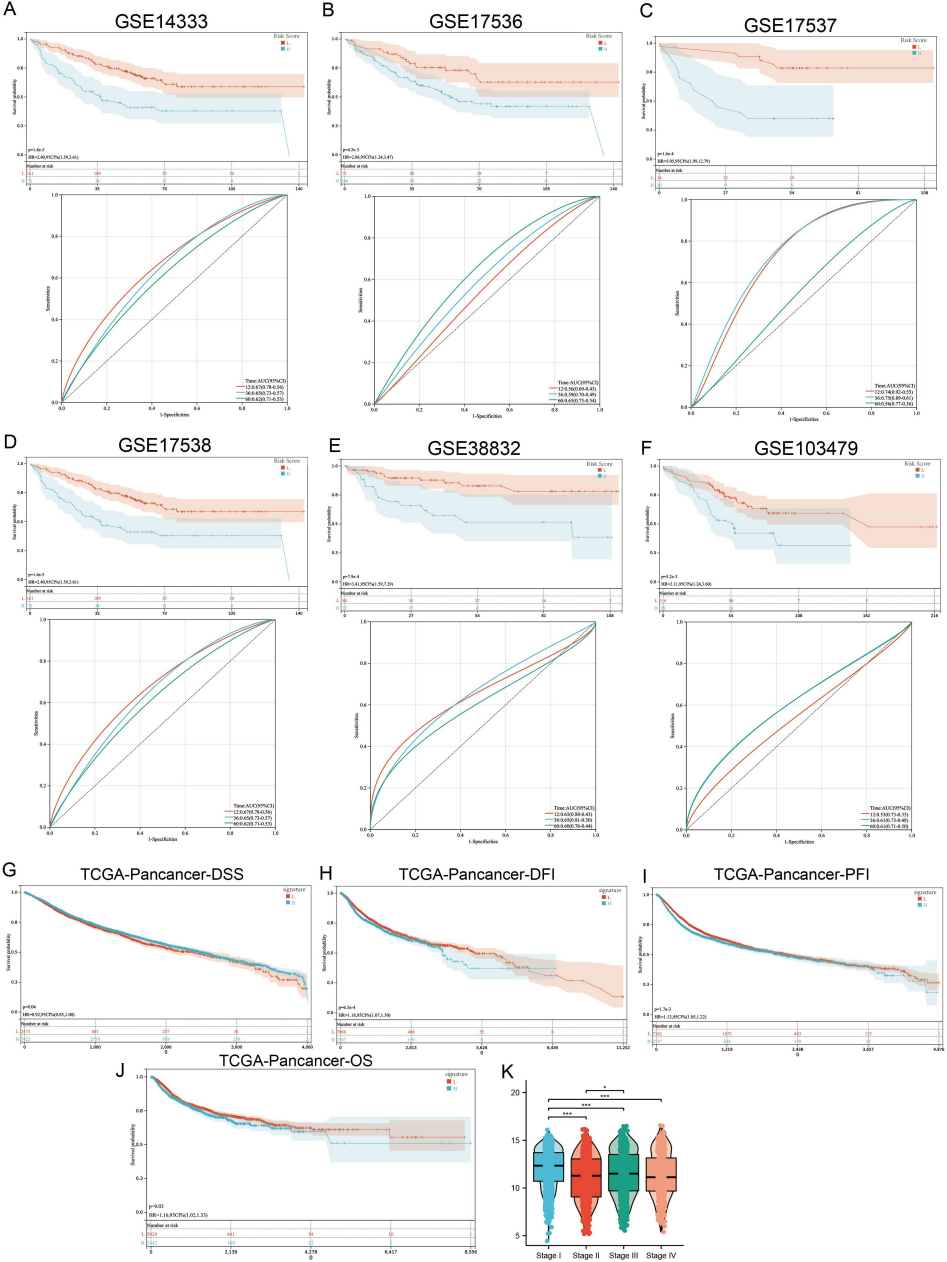
MPG’s Signature value in pan-cancer cohort. **(A–F)** 6 independent GSE cohorts affirmed that READ patients with higher signature score had poorer prognosis. **(G–J)** Patients with higher MPGS had poorer DFI, DSS, PFI, OS. **(K)** Signature score varies between different stages. *p < 0.05; ***p < 0.001 compared to the corresponding groups.

To extend the macrophage polarization gene signature (MPGS) ‘s prognostic utility, we assessed its performance in five additional GEO datasets containing survival information. Patients with higher MPGS-derived risk scores exhibited significantly worse overall survival in all cohorts. ROC curve analyses confirmed consistent predictive performance (**Figures 6A–F**).

Subsequently, we applied the MPGS to the TCGA pan-cancer dataset (n >11,000; 33 cancer types). Higher MPGS scores were significantly associated with worse disease-free survival (DFS), disease-specific survival (DSS), progression-free interval (PFI), and overall survival (OS) (**Figures 6G–J**). Furthermore, MPGS risk scores varied significantly across clinical stages (**Figure 6K**).

When evaluating individual cancer types, a hazard ratio (HR) > 1 for MPGS was observed in eight malignancies—BLCA, COAD, GBM, KIRC, LGG, LUSC, SKCM, and STAD—indicating a potential risk association. In contrast, MPGS was inversely associated with mortality (HR< 1) in 12 cancer types, suggesting a protective trend (**Supplementary Figure S5**).

### 3.7 Immune infiltration, mutation profiling, and PPI network analysis of hub genes

**Figure 7:**
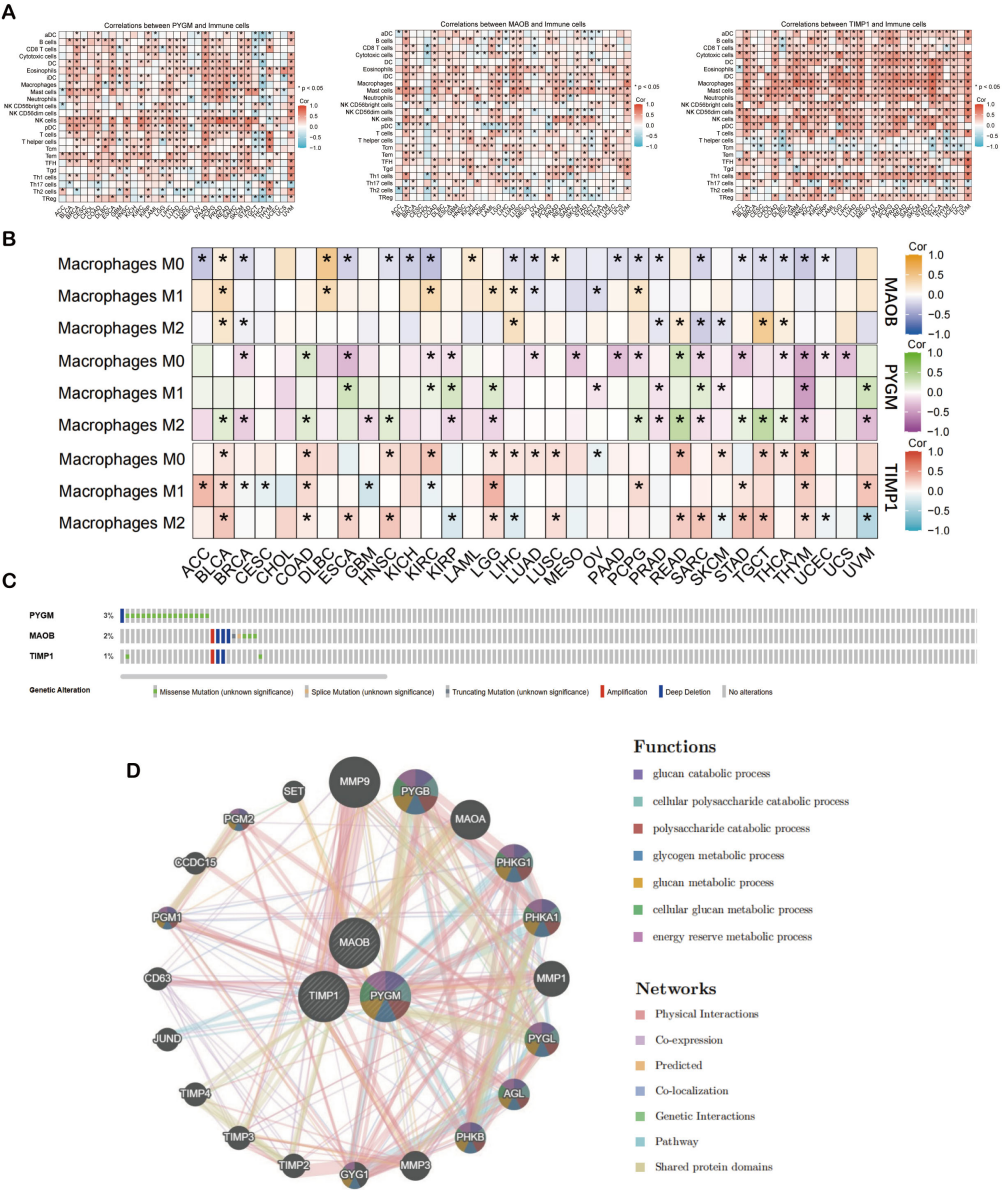
Analysis of prognostic macrophage polarization genes (MPGs). **(A)** Heat map of illustrating the result of ssGSEA algorithm of MPGs. **(B)** Heat map of illustrating the result of CIBERSORT algorithm of MPGs. **(C)** The mutation of MPGs in the cBioPortal database. The genetic alterations are represented by color coding. **(D)** The PPI network of the three hub genes from GeneMANIA database. *p < 0.05 compared to the corresponding groups.

To further investigate the immune associations of the three hub genes (*PYGM, MAOB, TIMP1*), we performed immune cell infiltration analyses across 33 TCGA cancer types using ssGSEA and CIBERSORT algorithms. ssGSEA showed a positive correlation between hub gene expression and macrophage infiltration in multiple cancer types, including BLCA, COAD, ESCA, HNSC, LGG, LUSC, PCPG, PRAD, READ, SKCM, and UVM (**Figure 7A**). CIBERSORT analysis further revealed that M2 macrophages exhibited a consistent positive correlation with hub gene expression in cancers such as BLCA, READ, and TGCT, while M1 macrophage correlation was limited, observed mainly in LGG (**Figure 7B**). *PYGM* expression was also positively associated with canonical M2 macrophage marker genes (**Supplementary Figure S6**).

Genomic profiling of MPGS genes using cBioPortal showed varying degrees of mutation frequency across cancer types (**Figure 7C**). Protein-protein interaction (PPI) analysis via GeneMANIA revealed that the hub genes, particularly *PYGM*, are functionally linked to glucose catabolism (**Figure 7D**). Matrix metalloproteinase 9 (MMP9) exhibited the strongest interaction, consistent with its established role in tumor invasion and metastasis (50).

### 3.8 Single-cell transcriptomic profiling of MPGs and metabolic associations

**Figure 8:**
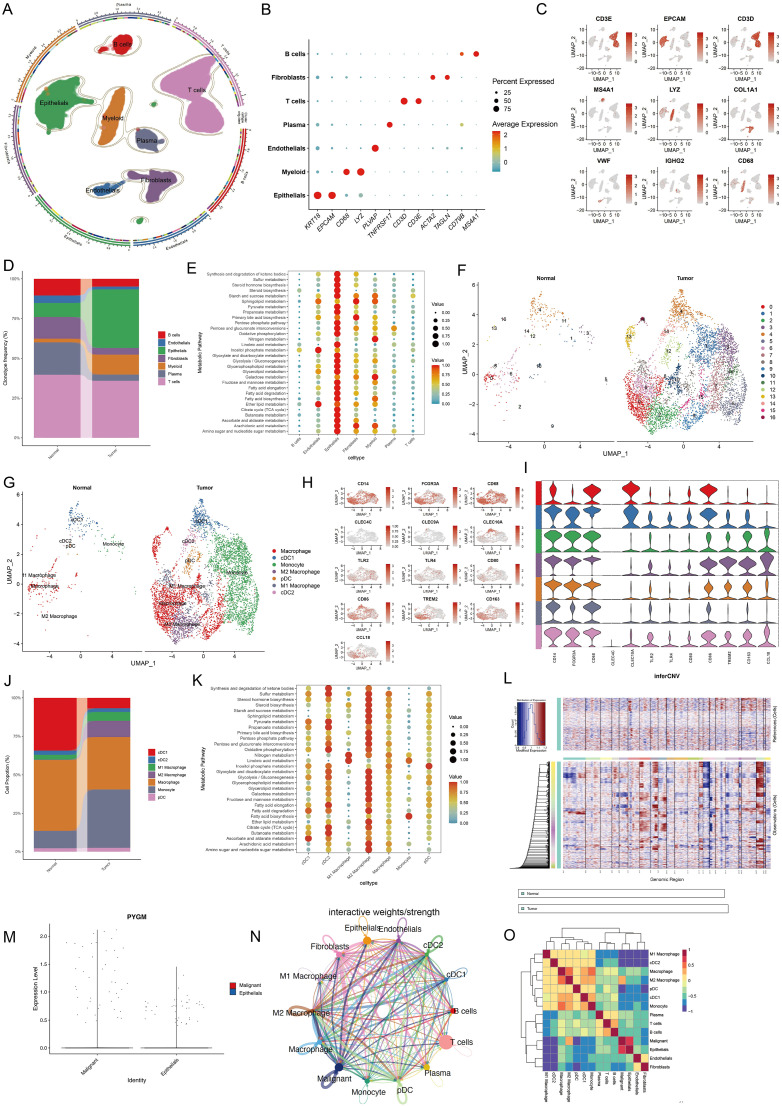
Single-cell transcriptomic analysis reveals cell type composition and metabolic hallmark signatures of TME in colorectal cancer. **(A)** UMAP showing subtypes of plasma cells, B cells, T cells, Epithelial cells, Myeloid cells Fibroblast cells and Endothelial cells. **(B, C)** Expression of marker genes used for the identification of each cluster. **(D)** Stacked bar plot representing the proportional distribution of cell types across different groups. **(E)** Dot plots showing average expression of known markers in indicated cell clusters. The dot size represents percentage of cells expressing the genes in each cluster. The expression intensity of markers is shown. **(F, G)** UMAP dimensionality reduction showing the integrated cell distribution map. A total of 16 cell clusters were identified, classified into 5 major cell types, with different colors representing distinct cell clusters. **(H, I)** Expression levels of selected known marker genes in UMAP plots from both normal and tumor tissue in CRC patients. **(J)** Stacked bar plot representing the proportional distribution of cell types across different groups. **(K)** Dot plots showing the enrichment of metabolic function in different cell types. **(L)** Chromosomal landscape of inferred CNVs among epithelial subclusters. **(M)** Violin plot showing the differential expression of PYGM between malignant and normal epithelial cells. **(N, O)** Cell-cell communication **(N)** and interaction analysis **(O)** revealed a strong association between malignant epithelial cells and M2 macrophages.

**Figure 9:**
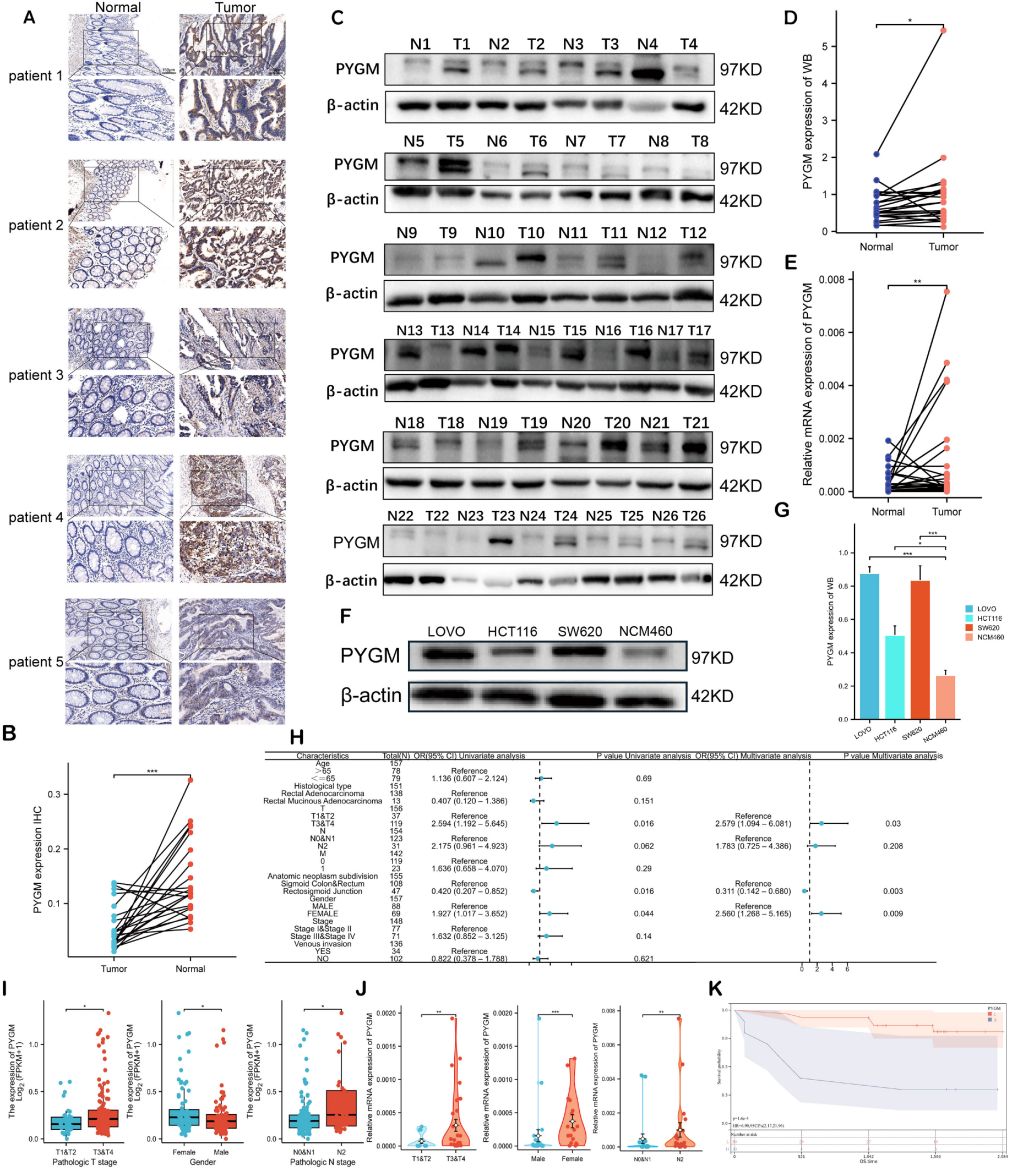
Validation of PYGM in clinical samples and cell line. **(A)** Immunohistochemical (IHC) results in rectal tumor and normal tissues. Original magnifications 15× and 40× (inset panels) **(B)** The expression levels PYGM in READ tissues (n = 21) and adjacent normal tissues (n = 21) from IHC. **(C, D)** WB assay of PYGM in READ tissues (n = 26) and adjacent normal tissues (n = 26). **(E)** mRNA expression levels of PYGM in paired samples of rectal cancer measured by qRT-PCR (n = 40). **(F, G)** WB assay of PYGM in different cell lines (HCT116, SW620, LOVO, NCM460). **(H)** Logistic regression analysis of PYGM in TCGA database. **(I)** Violin plots evaluating PYGM expression according to different clinical characteristics. **(J)** Violin plots evaluating PYGM expression of 40 clinical samples with READ according to different clinical characteristics. **(K)** OS curves between the high- and low-PYGM expression groups in the Yangpu Hospital cohort. *p< 0.05; **p< 0.01; ***p< 0.001 compared to the corresponding groups.

To examine the relationship between MPGs and tumor metabolism at single-cell resolution, scRNA-seq data from 23 rectal cancer patients and 10 healthy donors were analyzed. After integration and quality control, 63,689 cells were retained. Based on canonical markers, seven major cell types were identified: plasma cells (TNFRSF17), B cells (CD79B, MS4A1), T cells (CD3D, CD3E), epithelial cells (KRT18, EPCAM), myeloid cells (CD68, LYZ), fibroblasts (ACTA2, TAGLN), and endothelial cells (PLVAP) (**Figures 8A**, **9C**). Cell proportion analysis showed an increased abundance of epithelial and myeloid cells in tumor tissues (**Figure 8D**). Metabolic pathway enrichment analysis using hallmark gene sets from MSigDB revealed significant metabolic activation in epithelial cells, fibroblasts, and myeloid populations (**Figure 8E**).

Myeloid cells were further sub-clustered into 16 subsets and annotated into seven cell types, including M1/M2 macrophages, monocytes, cDC1, cDC2, and pDCs, based on marker expression and Spearman correlation with established cell-type profiles (**Figures 8F–I**). Tumor samples exhibited elevated M2 macrophages and monocytes (**Figure 8J**). Pathway analysis indicated significant metabolic enrichment—including glycolysis, fatty acid metabolism, and oxidative phosphorylation—in M2 macrophages (**Figure 8K**).

Given the significant enrichment of metabolic pathways in the epithelial cell subcluster, we applied the “inferCNV” R package to analyze this subcluster (**Figure 8L**), distinguishing malignant from normal epithelial populations. The results revealed that *PYGM* expression was markedly elevated in malignant epithelial cells (**Figure 8M**). Subsequent analyses of cell-cell communication and intercellular interactions demonstrated a strong association between malignant cells and M2 macrophages (**Figures 8N, O**). These findings suggest that *PYGM*, as a metabolism-related gene predominantly expressed by malignant epithelial cells, may play a regulatory role in modulating M2 macrophage activity within the tumor immune microenvironment, thereby contributing to tumor progression.

### 3.9 Experimental validation of hub gene expression in clinical and cellular models

Due to the prominent biological functions and immunotherapy-specific relevance of *PYGM*, it was selected for further validation. Immunohistochemistry (n = 21), Western blotting (n = 26) and qRT-PCR(n=40) further confirmed increased *PYGM* protein expression in READ tissues compared to adjacent normal tissues (**Figures 9A–D**). *In vitro*, assays also demonstrated higher *PYGM* expression in colorectal cancer cell lines (HT29, SW620, LOVO) compared to normal epithelial cells (NCM460) (**Figures 9F, G**). Besides, the expression patterns of MAOB and TIMP1 were determined using qRT-PCR analysis of clinical samples and HPA database (**Supplementary Figure S7A, B**).

Logistic regression analysis revealed that elevated *PYGM* expression was significantly associated with advanced T stage (p = 0.03), tumor anatomical subdivision (p = 0.003), and male sex (p = 0.009) (**Figure 9H**). TCGA and clinical validation cohorts confirmed higher *PYGM* levels in patients with advanced stage and males, which was inconsistent in patients in COAD (**Figures 9I–J**, **Supplementary Figure S7C**). Survival analysis further validated that higher *PYGM* expression was associated with poor OS (**Figure 9K**).

### 3.10 PYGM regulates CRC cell proliferation, apoptosis, migration and M2 macrophage polarization

**Figure 10:**
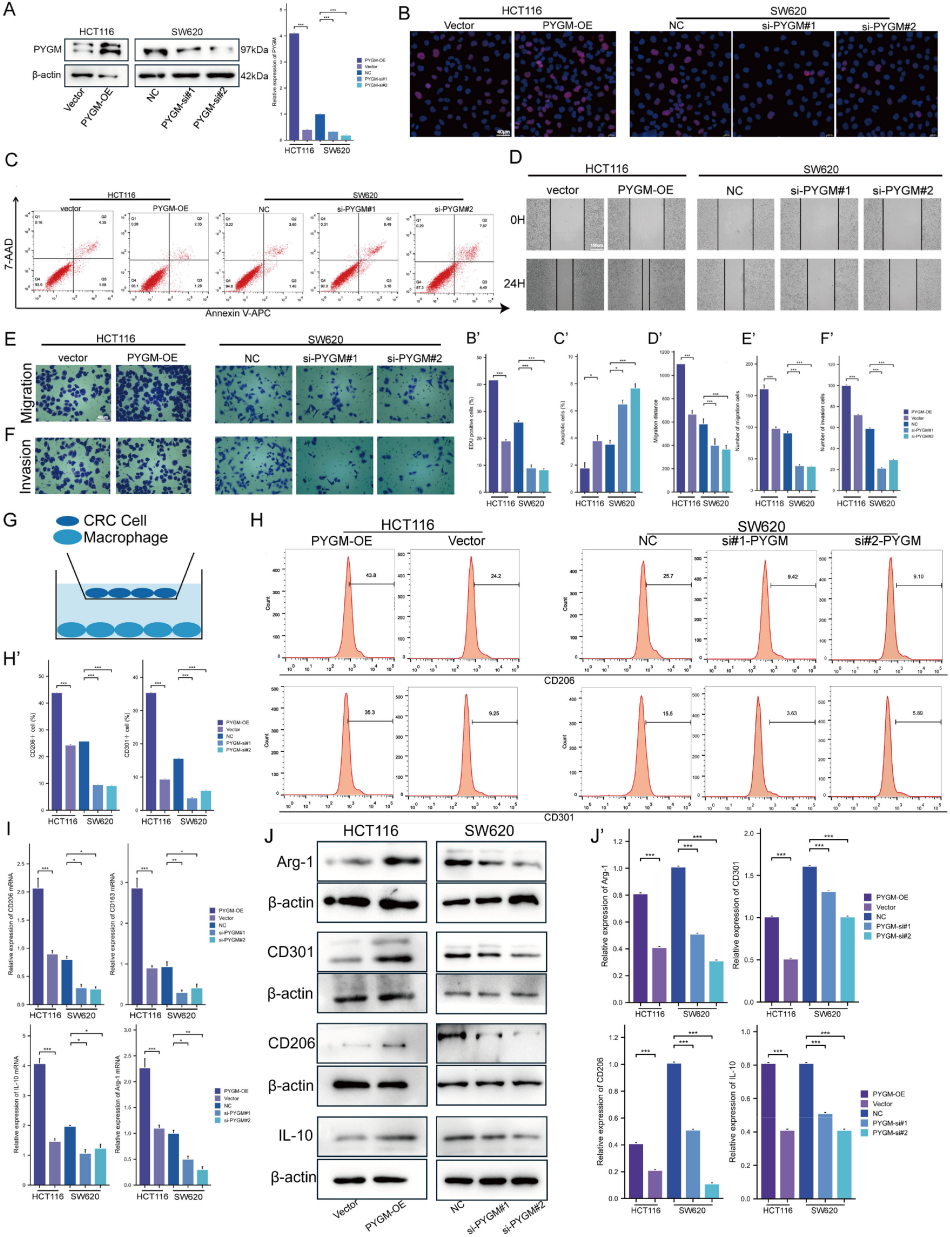
PYGM promotes colorectal cancer cell proliferation, migration, invasion, and M2 macrophage polarization. **(A)** Protein expression levels of PYGM were detected by western blotting. **(B)** EDU assay analysis. **(C)** Apoptosis rate was detected by flow cytometry. **(D–F)** Migration and invasion assay analysis. **(G)** Schematic illustration of the indirect co-culture system between colorectal cancer cells and THP-1-derived macrophages. **(H)** The expression levels of CD206 and CD301 were determined by flow cytometry. **(I)** Arg1, CD206, CD301 and IL-10 gene expression levels were detected by RT-PCR. **(J)** The protein levels of Arg1, CD206, CD301 and IL-10 were detected by western blotting. *p< 0.05; **p< 0.01; ***p< 0.001, compared to the corresponding groups.

In order to explore the functions of *PYGM* in RC, it was knocked down by siRNA in SW620 and overexpressed in HCT116, and the efficiency was verified by western blotting (**Figure 10A**).

EDU results showed that SW620 had a decreased proliferation, while HCT116 had an increased viability (**Figure 10B**). Furthermore, we detected the effect of *PYGM* on CRC apoptosis, and our study indicated that overexpression of *PYGM* significantly reduced the apoptosis rate of CRC cells, while knockdown of *PYGM* significantly increased the apoptosis rate of CRC cells (**Figure 10C**). The wound healing assay showed a marked decrease in cell migration following *PYGM* knockdown (**Figure 10D**) and an increase after its overexpression. Consistent with the results, transwell assays verified that *PYGM* knockdown inhibited SW620 invasion and migration, and its overexpression in HCT116 had the opposite trend (**Figures 10E, F**). THP-1 monocytes were induced into M2 macrophages using PMA and IL-4. PYGM-overexpressing or -silenced HCT116 and SW620 cells were co-cultured with these macrophages (**Figure 10G**). Flow cytometry revealed that CD206 and CD301 expression were elevated following *PYGM* overexpression, but reduced upon *PYGM* knockdown (**Figure 10H**). Similarly, RT-PCR showed corresponding changes in Arg1, CD206, CD301, and IL-10 mRNA levels in THP-1-derived macrophages (**Figure 10I**), which was further validated by western blotting (**Figure 10J**).

## 4 Discussion

Rectal cancer (RC) remains a biologically aggressive malignancy with limited treatment efficacy and high recurrence rates. Despite advancements in multimodal therapy, the molecular mechanisms underlying RC progression and therapeutic resistance remain incompletely understood. This study established a macrophage polarization gene signature (MPGS) using integrative bioinformatic and machine-learning approaches. The signature comprises three macrophage-related genes—*TIMP1*, *MAOB*, and *PYGM*—that robustly stratify rectal adenocarcinoma (READ) patients by prognosis. By linking tumor-associated macrophage (TAM) infiltration with metabolic remodeling and clinical outcomes, our findings provide a framework for precision oncology strategies targeting the tumor immune microenvironment.

Macrophages are integral components of the tumor microenvironment (TME), exhibiting functional plasticity that supports tumor progression. In particular, M2-polarized macrophages have been associated with immune suppression, angiogenesis, and metastasis in various solid tumors, including ovarian, breast, and gastric cancers (12, 17, 22, 51). Our study corroborated these findings by demonstrating a significant association between M2 macrophage infiltration and poor prognosis in READ. These results underscore the importance of immunological context in shaping disease trajectory and therapeutic response.

We employed a robust modeling strategy incorporating WGCNA and four machine learning algorithms (LASSO, SVM-RFE, RF, and XGBoost) to construct a reliable prognostic tool. This multi-algorithmic framework mitigated overfitting and bias, ensuring that gene selection was statistically and biologically grounded. The final MPGS was independently validated in multiple datasets, demonstrating strong predictive performance. Integrating machine learning and biological relevance positions MPGS as a clinically applicable model for outcome prediction in RC.

Among the three genes comprising MPG, *PYGM* emerged as a particularly compelling candidate due to its metabolic functions and correlation with poor prognosis. While *PYGM* is classically known for its role in glycogen metabolism in skeletal muscle (52), recent studies have implicated it in oncogenic metabolic pathways in gastric, renal, breast, and head and neck cancers (53–56). However, its immunological relevance has remained largely unexplored. Our findings provide novel evidence linking *PYGM* expression with M2 macrophage enrichment and immunosuppressive phenotypes in the TME.

MPGS demonstrated strong prognostic performance across both training and external validation cohorts. Higher risk scores were consistently associated with worse overall survival (OS), as shown through Kaplan–Meier and ROC analyses. MPGS retained independent prognostic value in multivariate Cox models after adjusting for conventional clinical variables. These results indicate that MPGS may complement current staging systems and provide additional prognostic stratification in clinical settings.

Functional enrichment analyses revealed that MPGS-related genes are involved in immune-related and oncogenic pathways, including MAPK, TGF-β, Jak-STAT, and PI3K-AKT signaling (57, 58). These pathways are well-documented mediators of macrophage polarization and tumor progression. For instance, activation of STAT3 promotes M2 macrophage differentiation and secretion of immunosuppressive cytokines (59). Likewise, the TGF-β and PI3K-AKT pathways have been shown to facilitate tumor angiogenesis and resistance to anti-angiogenic therapy through macrophage reprogramming (60–62). These findings support the hypothesis that MPGS reflects the immunometabolic landscape of the TME.

Immune infiltration analysis further validated the relevance of MPGS. High-risk patients exhibited increased infiltration of M2 macrophages and lower immune and stromal scores, indicative of an immunosuppressive microenvironment. ssGSEA and CIBERSORT analyses across multiple cancer types confirmed that the three core genes are positively associated with macrophage-mediated immunosuppression, particularly through M2 polarization. These results suggest that MPGS may be a predictive biomarker for immunotherapeutic response in RC and potentially in other malignancies.

The MPGS model also demonstrated predictive utility for drug sensitivity. Patients with higher MPGS scores tend to exhibit poorer responses to immunotherapy and worse clinical outcomes. Patients showed differential responses to several chemotherapeutic agents with different score. These findings imply that MPGS may inform therapeutic decision-making, enabling clinicians to tailor chemotherapeutic and immunotherapy regimens based on an individual’s macrophage polarization profile and risk classification.

Beyond READ, MPGS was evaluated in multiple independent GEO datasets and TCGA pan-cancer cohorts. High-risk scores consistently predicted poorer outcomes across various tumor types, including bladder cancer, glioblastoma, and gastric cancer. These results support the generalizability of MPGS and reinforce the notion that TAM-mediated immunosuppression is a common pathological feature across cancers.

Among the three MPGS genes, *PYGM* was selected for further experimental validation due to its strong prognostic significance, central metabolic role in the PPI network and strongest relationship with M2 macrophage. Our single-cell transcriptomic analysis indicates that malignant epithelial cells with elevated *PYGM* expression display enhanced metabolic activity and engage in frequent crosstalk with M2 macrophages. This suggests that *PYGM* may actively contribute to establishing an immunosuppressive tumor microenvironment by promoting M2 macrophage polarization, ultimately facilitating tumor progression. It is well established that distinct activation states of macrophages are accompanied by profound intracellular metabolic reprogramming, including glycolysis, oxidative and lipid metabolism (63, 64). CD36 serves as a fatty-acid translocase on immune cells, modulating lipid uptake in Tregs, CD8^+^ T cells and macrophages, and reshaping immune responses through autophagy/FAO pathways (28). Similarly, SREBPs predominantly regulate lipid biosynthesis, and could influence immunometabolic processes via AKT/mTORC1/GPX4 signaling pathway, affecting T-cell and macrophage functions (65). In contrast, PYGM is a key enzyme in glycogen catabolism, converting glycogen into glucose-1-phosphate (53). Its interaction with metabolic regulators, including AMPK, suggests a novel axis for modulating macrophage and myeloid immune cell function in the TME (66, 67). This complements but differs mechanistically from SREBP/CD36 pathways that center on lipid metabolism.

The expression of *PYGM* was elevated in tumor tissues and cell lines and significantly correlated with advanced tumor stages, anatomical location, sex and poor prognosis. Moreover, a series of cellular assays confirmed that *PYGM* enhances tumor cell proliferation, inhibits apoptosis, and promotes migration and invasion. In addition, *PYGM* was found to facilitate macrophage polarization toward the M2 phenotype.

Despite the robustness of our computational and experimental findings, this study has limitations. Most notably, the molecular mechanisms by which *PYGM* and the other MPGS genes modulate TAM behavior remain to be elucidated. Further *in vivo* and mechanistic studies are warranted to clarify the causal relationships between *PYGM* expression and tumor progression in RC.

In summary, we present a validated macrophage polarization gene signature that effectively stratifies patients with rectal adenocarcinoma and correlates with immunosuppressive TME features. Among its components, *PYGM* emerged as a promising metabolic and immunologic biomarker with prognostic and potential therapeutic relevance. These findings expand our understanding of macrophage-driven tumor progression and lay the groundwork for clinical strategies integrating immunometabolic profiling into precision oncology.

## Data Availability

The original contributions presented in the study are included in the article/**Supplementary Material**, further inquiries can be directed to the corresponding author/s.

## Glossary

OS: overall survival
ROC: receiver operating characteristic
GSEA: gene get enrichment analysis
MPGS: macrophage polarization gene signature
DEGs: differentially expressed genes
qRT-PCR: quantitative real-time polymerase chain reaction
IHC: immunohistochemistry
WB: western blotting
ROC: receiver operating characteristic curves
TME: total mesorectal excision
LASSO: least absolute shrinkage and selection operator
SVM: support vector machine
RFE: recursive feature elimination
RF: random forest
XGBoost: eXtreme gradient boosting
WGCNA: weighted gene co-expression network analysis
MPGs: macrophage polarization genes
READ: rectal adenocarcinoma
HR: hazard ratio
nCRT: neoadjuvant chemoradiotherapy
TAMs: Tumor-associated macrophages
GO: Gene ontology
KEGG: Kyoto Encyclopedia of Genes and Genomes
FDR: false discovery rate
ssGSEA: single-sample GSEA
PPI: Protein-protein interaction
IC50: Half-maximal inhibitory concentration
TIDE: Tumor Immune Dysfunction and Exclusion
TCIA: The Cancer Immunome Atlas
