# Supplementary material for: Machine learning identifies PYGM as a macrophage polarization–linked metabolic biomarker in rectal cancer prognosis
Source: Front Immunol. 2025 Aug 12;16:1639303. doi: 10.3389/fimmu.2025.1639303 (PMC12378483; doi:10.3389/fimmu.2025.1639303)
Supplement: Supplementary file 1 [file Table1.docx]

**Supplementary table 1**

|  | Forward | Reverse |
| --- | --- | --- |
| PYGM | 5'-CCATGCCCTACGATACGCC-3' | 5'-TAGCCACCGACATTGAAGTCC-3' |
| MAOB | 5'-GGACAACATGACAATGAA  GGA-3' | 5'-TGACTGAACCCAAAGGCAC  AC-3' |
| TIMP1 | 5'- CTTCTGCAATTCCGACCTCGT-3' | 5'- ACGCTGGTATAAGGTGGTCTG-3' |
| Arg-1 | 5’CCCTGGGGAACACTACATTTTG-3’ | 5’-GCCAATTCCTAGTCTGTCCACTT-3’ |
| β-actin | 5'-CCTGGCACCCAGCACAAT-3' | 5'-GGGCCGGACTGTCATAC-3' |
| CD206 | 5’- CTACAAGGGATCGGGTTT  ATGGA-3’ | 5’- TTGGCATTGCCTAGTAGCGTA-3’ |
| IL-10 | 5’- TCAAGGCGCATGTGAACTCC-3’ | 5’- GATGTCAAACTCACTCATGGCT-3’ |
| CD301 | 5’-GCGGGAGAGTGGAAGTGAAG-3’ | 5’-GTTACAAATCACAGAGACCGCT-3’ |

**Supplementary table 2**

| Reagent Type | Name / ID | Sequence / Product Information |
| --- | --- | --- |
| siRNA (Knockdown) | siPYGM-A1 | Sense: 5′-GCUAUUGCUAGGACUUUGA dTdT-3′ Antisense: 5′-UUC AAGUCCUAGCAAUAG C dTdT-3′ |
| siRNA (Knockdown) | siPYGM-A2 | Sense: 5′-CAGAUAAGGAUUUCAAGAA dTdT-3′ Antisense: 5′-UUC UUGAAAUCCUUAUCUG dTdT-3′ |
| Plasmid (Overexpression) | PYGM overexpression clone | Full-length human PYGM ORF (NM_005609.4), mGFP-DDK tagged, Lenti vector, sequence-verified |


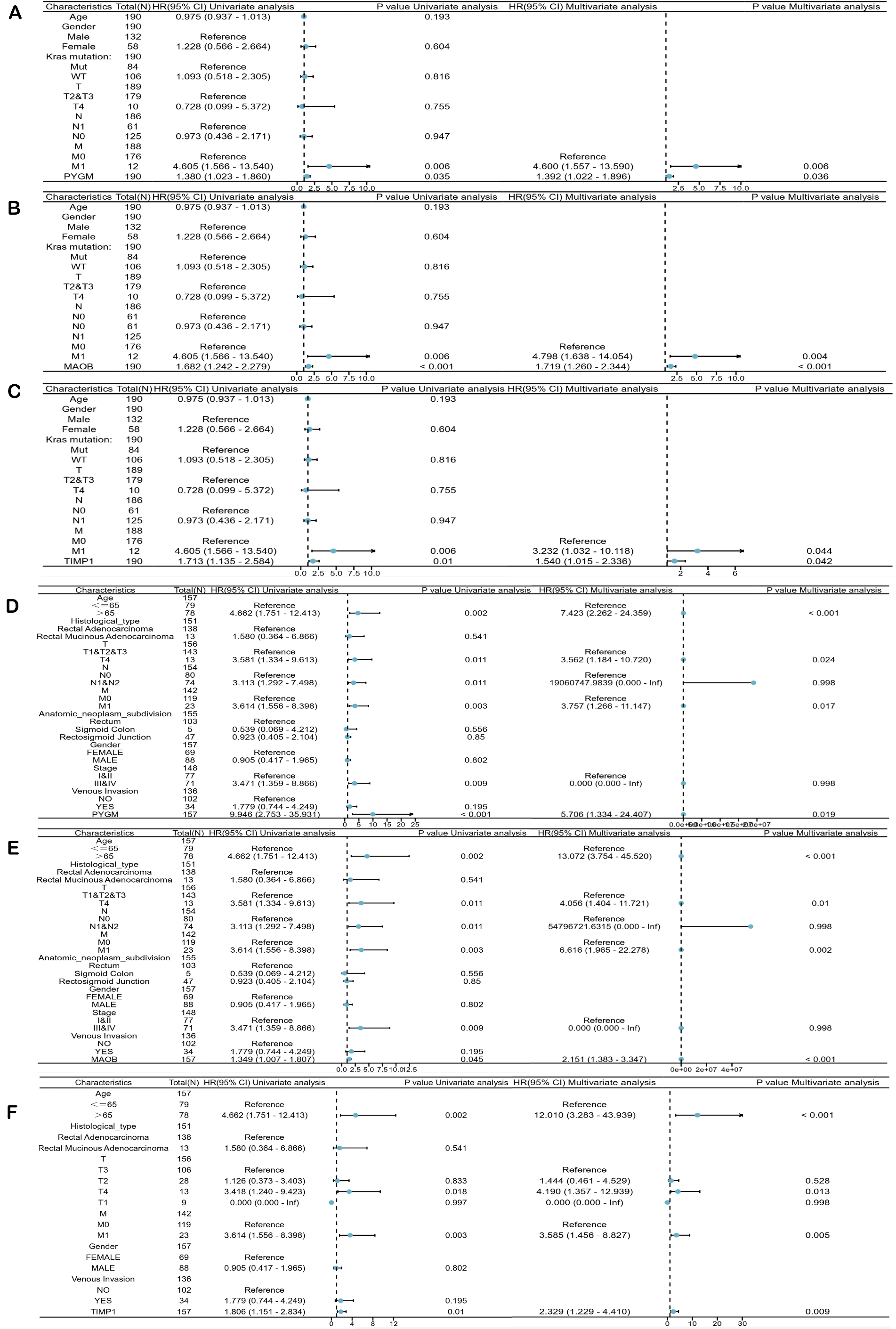


**Figure S1** Univariate and multivariate Cox regression analyses of PYGM, MAOB and TIMP1 (**A-C**) Uni- and multivariate Cox analyses of PYGM, MAOB, TMIP1 expression and other clinical pathological variables in GSE87211. (**D-F**) Uni- and multivariate Cox analyses of PYGM, MAOB, TMIP1 expression and other clinical pathological variables in TCGA-READ

| 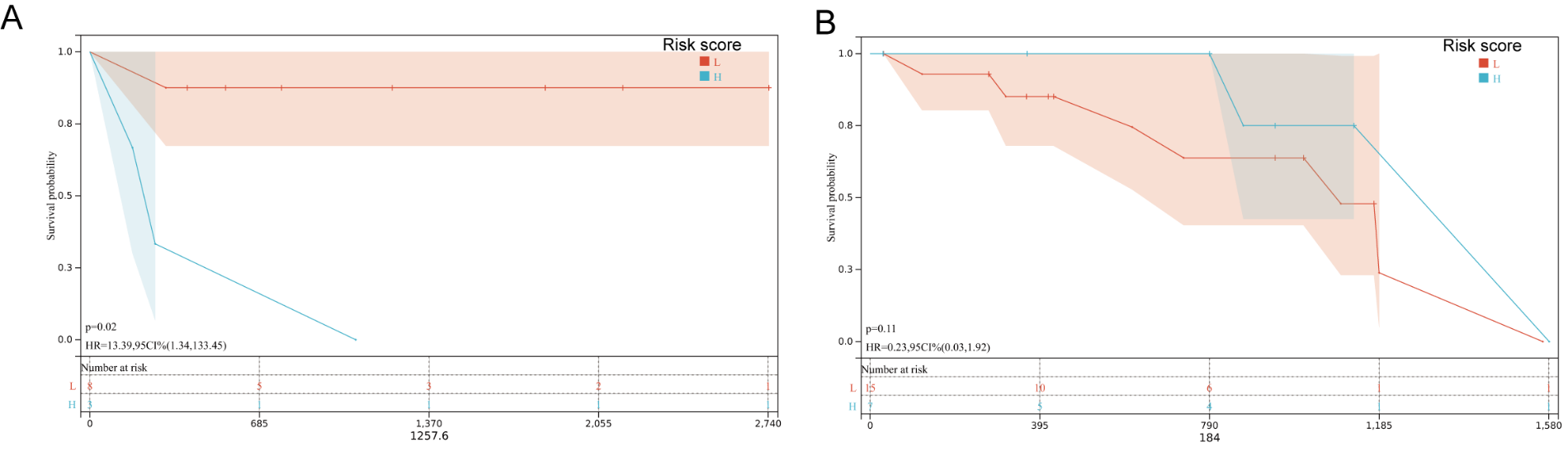 |
| --- |
| **Figure S2** Survival curves of MPGS in M1 subgroups in GSE87211 **(A)** and TCGA **(B)** datasets. |

| 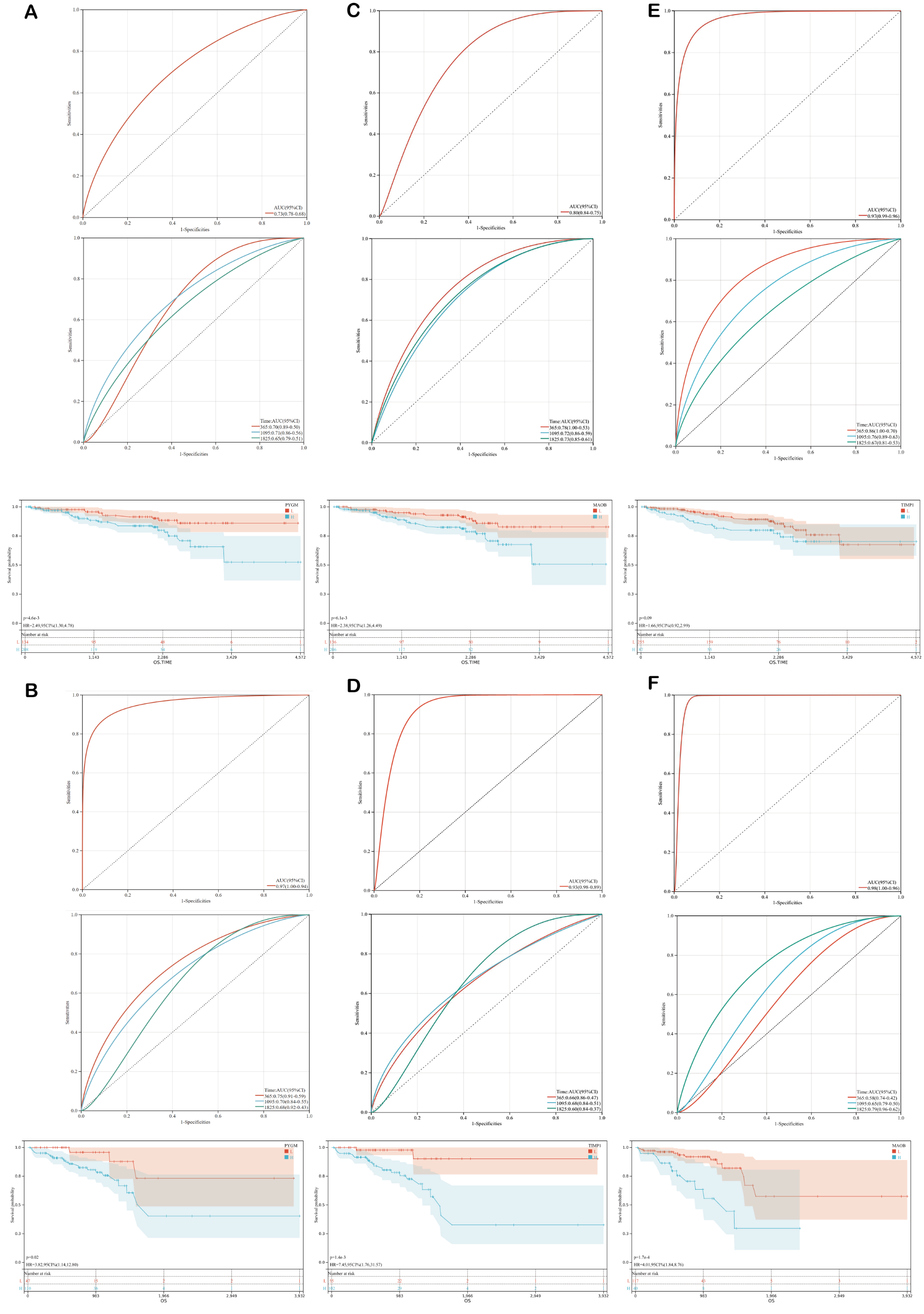 |
| --- |
| **Figure S3** Diagnostic ROC curves, time-dependent ROC analysis and survival curves of PYGM **(A-B)**, MAOB **(C-D)**, TIMP1 **(E-F)** in GSE87211 and TCGA cohort. |


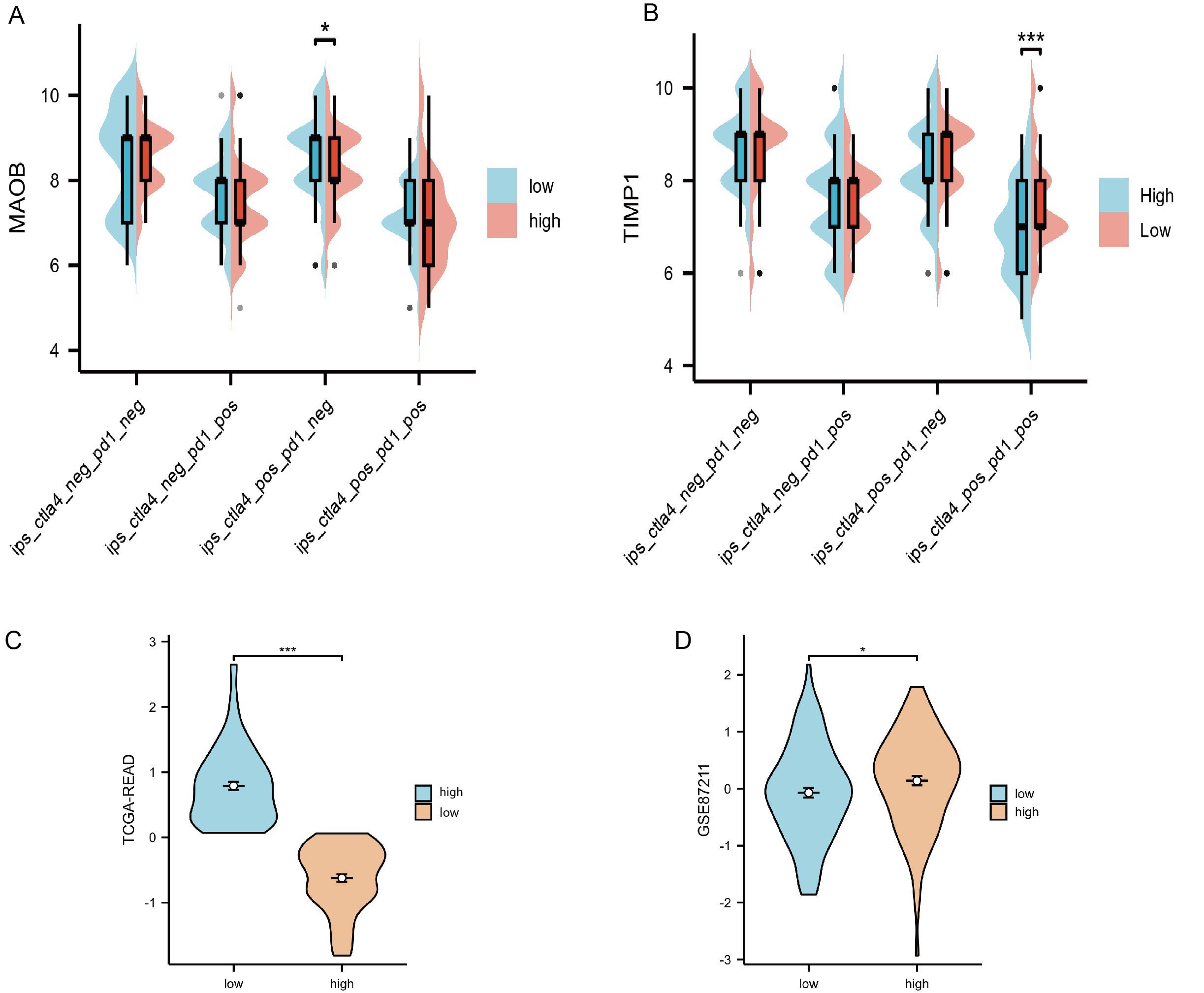


**Figure S4: (A-B)** Correlation of IPS with MAOB and TIMP1 expression. **(C-D)** Correlation of PYGM expression with TIDE. *p < 0.05; **p < 0.01; ***p < 0.001 compared to the corresponding groups.


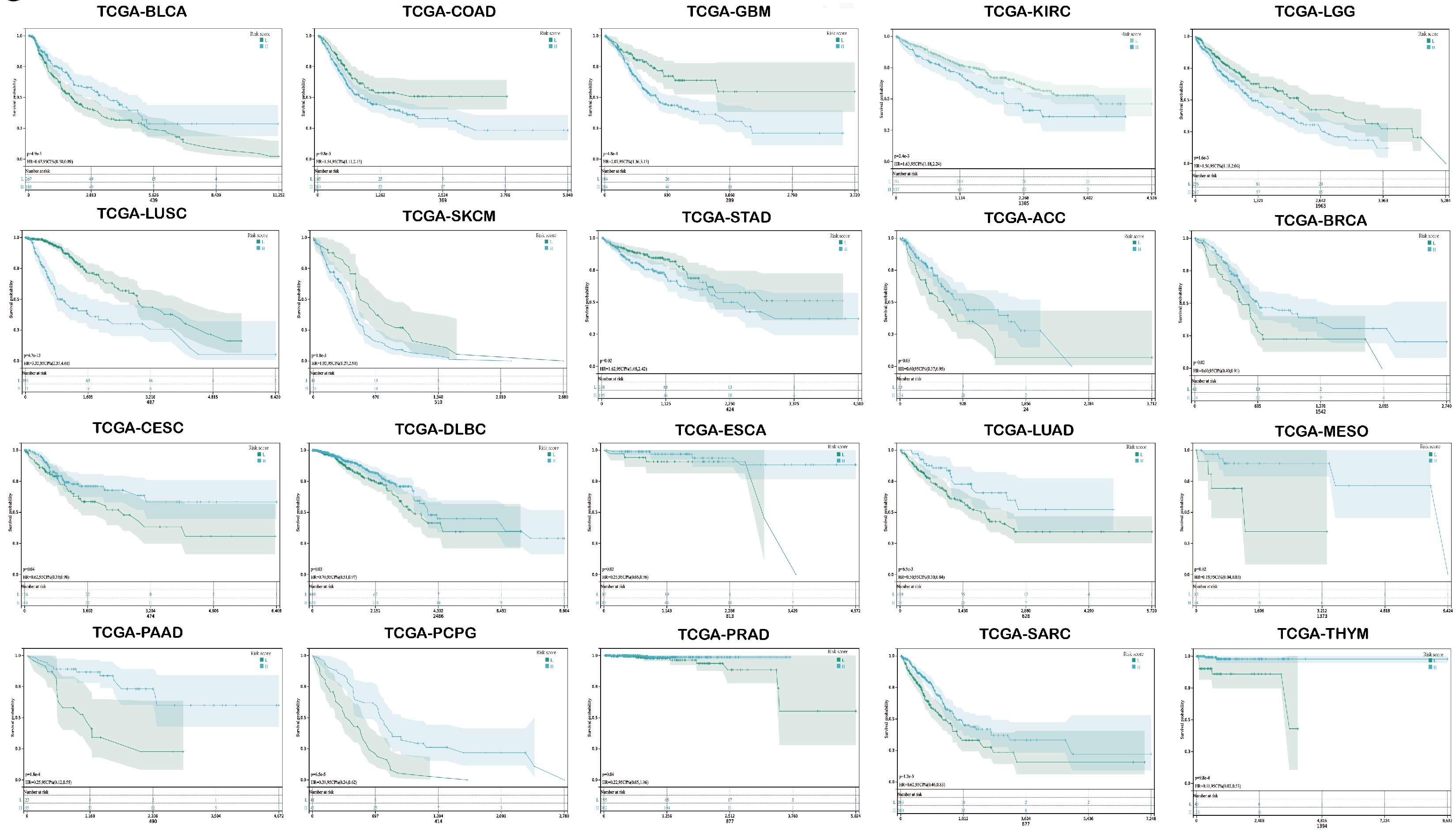


**Figure S5** MPGS is a risk factor for 8 types of cancer (BLCA, COAD, GBM, KIRC, LGG, LUSC, SKCM, STAD) while protective factor for other 12 types (ACC, BRCA, CESC, DLBC, ESCA, LUAD, MESO, PAAD, PCPG, PRAD, SARC, THYM).

| 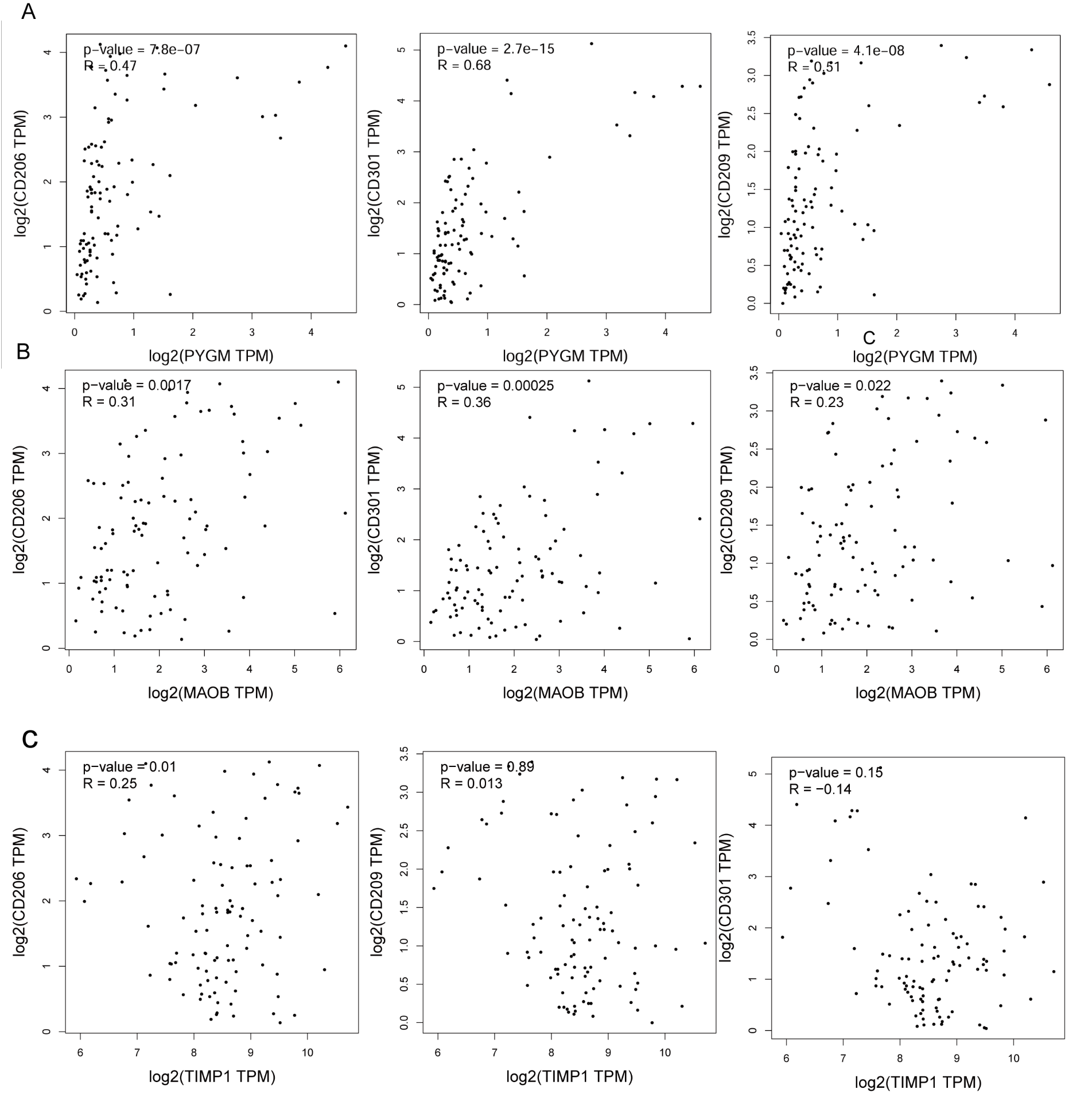 |
| --- |
| **Figure S6** Correlation analysis between PYGM **(A)**, MAOB **(B)**, TIMP1 **(C)** expression and M2 Macrophage markers (CD206, CD209, CD301). |


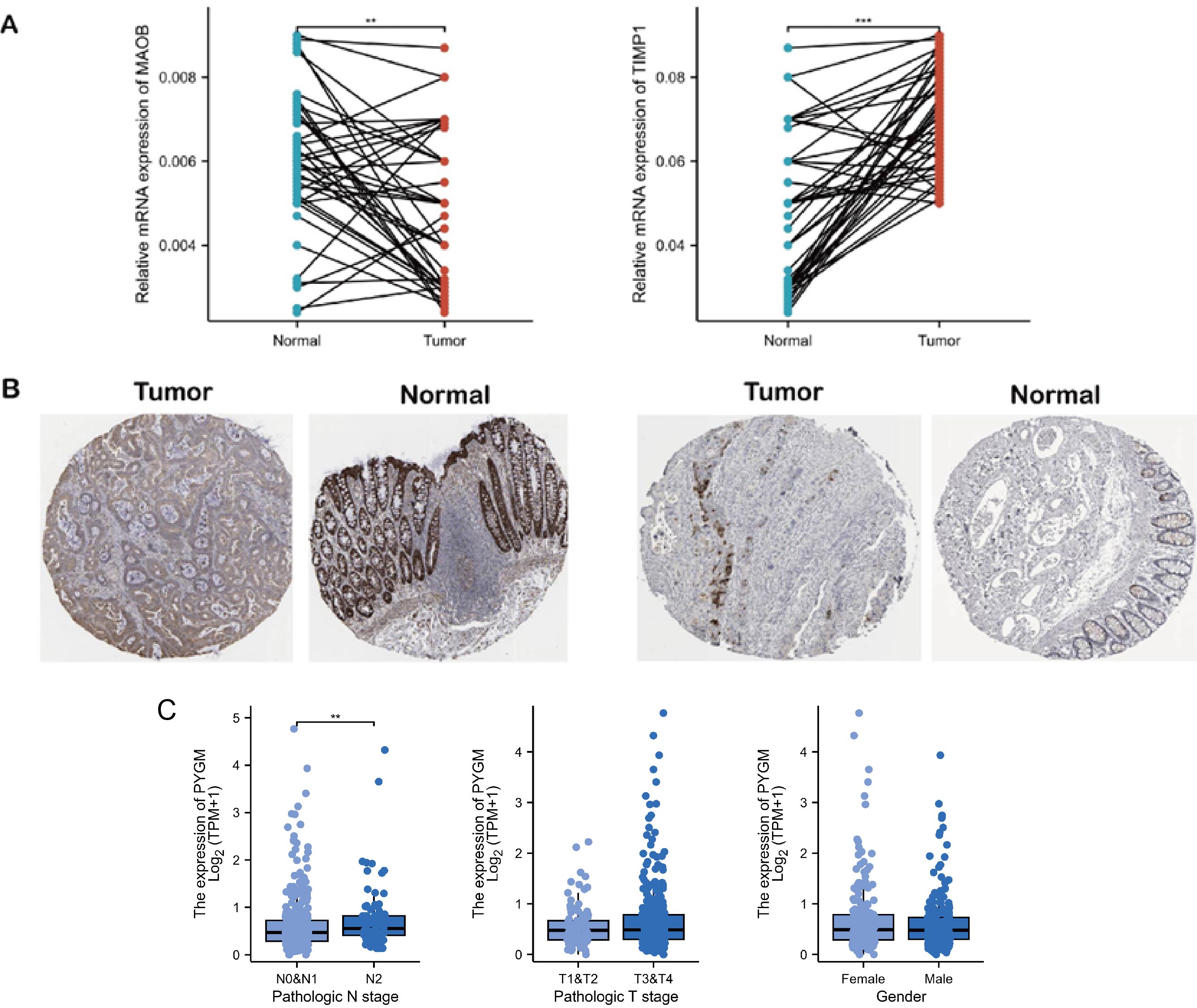


**Figure S7** Differential expression of MAOB and TIMP1 in RT-qPCR (n=40) **(A)** and HPA database **(B)**. **(C)**The expression patterns of PYGM in clinical subgroups of N stage, T stage, gender in TCGA-COAD. **p < 0.01; ***p < 0.001 compared to the corresponding groups.
